# Interplay between ALK2^R206H^ mutant receptor and autophagy signaling regulates receptor stability and its chondrogenic functions

**DOI:** 10.1038/s41420-025-02393-0

**Published:** 2025-03-22

**Authors:** Laura Coculo, Marius Wits, Irene Mariani, Giulia Fianco, Serena Cappato, Renata Bocciardi, Nicoletta Pedemonte, Elisabetta Volpe, Serena Ciolfi, Rosario Luigi Sessa, Serena Rinaldo, Francesca Cutruzzolà, Daniela Trisciuoglio, Marie-Josè Goumans, Gonzalo Sanchez-Duffhues, Venturina Stagni

**Affiliations:** 1 Institute of Molecular Biology and Pathology, National Research Council (CNR), Rome, Italy; 2 Cell Signalling Unit, Istituto di Ricovero e Cura a Carattere Scientifico (IRCCS), Rome, Italy; 3 Department of Cell & Chemical Biology, Leiden University Medical Center (LUMC), Leiden, The Netherlands; 4 UOC Genetica Medica, IRCCS Istituto Giannina Gaslini, Genoa, Italy; 5 DINOGMI, University of Genoa, Genoa, Italy; 6 Molecular Neuroimmunology Unit, IRCCS Fondazione Santa Lucia, Rome, Italy; 7 Department of Biochemical Sciences, Sapienza University of Rome, Rome, Italy; 8 Nanomaterials and Nanotechnology Research Center (CINN), Spanish National Research Council (CSIC), Health Research Institute of Asturias (ISPA), Oviedo, Asturias Spain

**Keywords:** Macroautophagy, Growth factor signalling

## Abstract

Heterozygous mutations in the Bone morphogenetic protein (BMP) type I receptor *ACVR1*, encoding activin-like kinase 2 (ALK2), underlie all cases of the rare genetic musculoskeletal disorder Fibrodysplasia Ossificans Progressiva (FOP). The most commonly found mutant ALK2 p.R206H receptor variant exhibits loss of auto inhibition of BMP signaling and can be activated by Activins, while wild-type receptors remain unresponsive. Consequently, the downstream chondrogenic signaling is enhanced, thus driving heterotopic ossification within soft connective tissues. Despite several investigational treatments being evaluated in clinical trials, no cure for FOP exists today. The cellular and molecular mechanisms underlying disease progression are still being deciphered. In this study, we show a close interplay between the mutant ALK2^R206H^ receptor signaling and dysregulation of the autophagic flux triggered by hypoxia. Mechanistically, reduced autophagic flux correlates with increased stability of ALK2^R206H^, resulting in sustained signaling. Of note, we demonstrated that Rapamycin, under clinical investigation as a treatment for FOP, inhibits chondrogenic differentiation in an autophagy-dependent manner. Consistently, other pharmacological autophagy inducers, like Spermidine, can reduce ALK2^R206H^ driven chondrogenic differentiation in vitro. These results were verified in FOP patient-derived cells. In conclusion, this study shows that aberrant autophagic flux mediates sustained ALK2^R206H^ signaling, introducing a novel druggable target in FOP by reactivating autophagy.

## Introduction

Activin receptor-like kinase 2 (ALK2), encoded by the gene Activin receptor type I (ACVR1*)*, belongs to the transforming growth factor β (TGF-β) family receptors. ALK2 is a type I bone morphogenetic protein (BMPs) receptor that transduces extracellular signals in response to ligands, including BMP5, BMP6, BMP7, and, with lesser affinity, BMP9 [1, 2]. In addition, ALK2 can bind a non-BMP set of ligands named Activins. Upon BMP ligand binding, ALK2 partners with the constitutively active type II BMP receptors, including BMPRII and activin receptor type-2A (ACVRIIA), to form tetrameric complexes that mediate the activation of the intracellular canonical BMP mediators, the small mothers against decapentaplegic (SMAD) 1/5/8 [3, 4]. Given the key functions of BMPs, their activity is tightly regulated at different levels, from ligand and receptor bioavailability to the expression of target genes and proteins [5]. Aberrant behavior of components of the BMP pathway, particularly ALK2, has been associated with several human diseases, including Fibrodysplasia ossificans progressiva (FOP) [1, 2].

FOP is an ultra-rare genetic disorder (MIM 135100) mainly characterized by congenital malformed big toes and postnatal episodes of extra-skeletal bone formation (named heterotopic ossification, HO) of soft connective tissues through endochondral ossification, that is, bone emergence through the formation of a cartilage template that is eventually replaced with mature bone [6, 7]. HO is typically manifested within the first 5 years of life, leading to severely impaired mobility around the second and third decade of life. Patients’ death is often caused by cardiothoracic insufficiency syndrome. HO is induced by inflammatory lesions (so-called flare-ups), triggered by minor injuries or without detectable trauma [6, 7]. Currently, there is no widely available therapy for this devastating disease [8]. FOP is caused by a recurrent heterozygous gain-of-function mutation in ALK2 [9], and postnatal expression of human mutant ALK2 (ALK2^R206H^) in mice results in a FOP-like disease, supporting the central pathogenic role of aberrant ALK2 signaling in FOP [10]. The canonical FOP mutation (c.617G > A, p.R206H) leads to an increased activation of ALK2-mediated signaling, for which different underlying mechanisms have been shown. As such, it has been shown that ALK2^R206H^ exhibits loss of autoinhibition, resulting in a leaky activation, which correlates with enhanced chondrogenic differentiation [11]. Importantly, unlike the wild-type ALK2, the ALK2^R206H^ variant renders the receptor responsive to the pro-inflammatory cytokine Activin A [12, 13]. Activin A is considered a non-osteogenic ligand of the TGF-β superfamily and normally signals via ALK4 and ALK7 [14]. Besides this neo-functional response of the mutant ALK2 receptor, additional mechanisms may enhance mutant-dependent pathogenic signaling, including hypoxia and inflammation. The hypoxia inducible factor-1-α (HIF-1-α) has been reported to promote the amplification of BMP signaling through the retention of the mutated ALK2 in signaling endosomes [15, 16]. How aberrant ALK2^R206H^ signaling leads to HO, particularly in connective tissues affected in FOP, is an area of intense investigation [16, 17]. In this study, we further explored the crosstalk between the ALK2 receptor and other signaling pathways. We primarily focused on autophagy, a process triggered by microenvironmental factors within HO lesions, including hypoxia, reactive oxygen species formation, and inflammation [18].

Autophagy is a highly regulated catabolic process that involves sequestration and lysosomal degradation of cytosolic components, such as dysfunctional organelles, misfolded proteins, or other components to be recycled [19]. Autophagy is an essential pathway for muscle repair and cartilage homeostasis, where it supports chondrocyte expansion mediated by hypoxia in vivo [18]. Recently, using an in vivo zebrafish model, it has been demonstrated that autophagy is required during normal chondrogenesis by preventing early chondrocyte maturation [20] and chondrocyte homeostasis in hypoxia [21, 22]. Inflammation and hypoxia have been described to occur during the formation of HO lesions in FOP [15, 16]. In this phase, inhibition of the central regulator of the autophagic pathway mammalian target of rapamycin (mTOR) effectively suppressed HO. Accordingly, a clinical trial study is currently ongoing to evaluate the efficacy and safety of Rapamycin in FOP [16, 23].

Despite this suggestive evidence, to date, the role of autophagy in ALK2 signaling remains inconclusive. In this study, we demonstrate that the expression of ALK2^R206H^ induces a dysregulation of the autophagic flux that correlates with the stabilization of the mutant receptor in ATDC5 chondrogenic cells. We confirmed the beneficial effects of rapamycin in reducing chondrocyte differentiation in a FOP cellular model, where rapamycin reactivates autophagy in ALK2^R206H^ expressing cells. At the molecular level, we discovered a negative feedback loop through which ALK2^R206H^/SMAD1 are degraded through the lysosomal pathway upon autophagy flux reactivation. We have confirmed these data on Endothelial colony-forming cells (ECFCs) derived from FOP patients. Altogether, we demonstrated a novel molecular mechanism through which autophagy reactivation is beneficial by favoring degradation of the ALK2 ^R206H^ mutant receptor.

## Results

### Overexpression of ALK2^R206H^ hampers the autophagy pathway

**Fig. 1:**
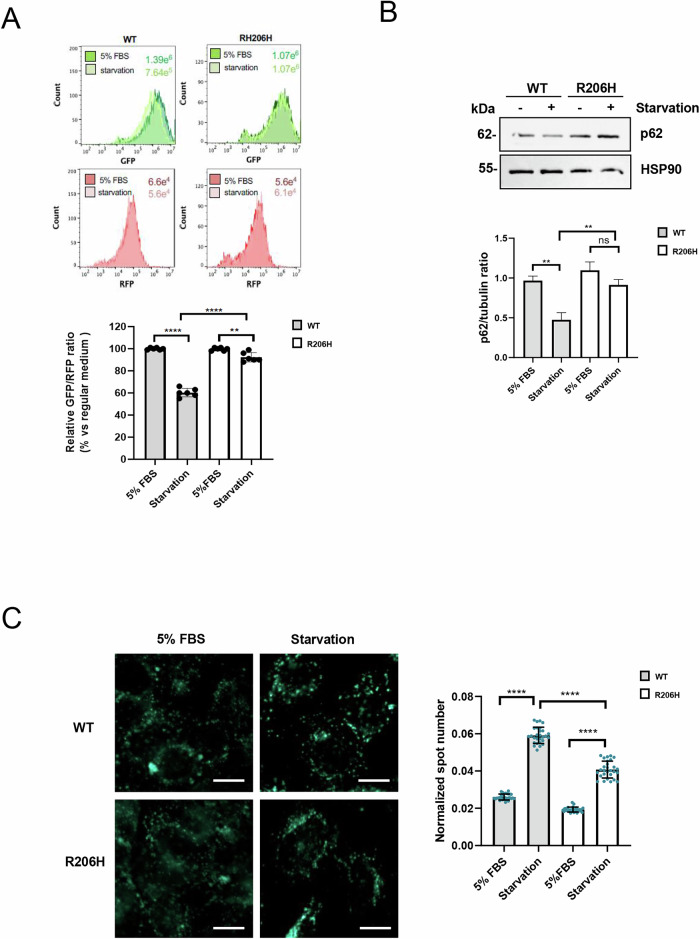
Overexpression of ALK2^R206H^ hampers the autophagy pathway. **A** Flow cytometry analysis in ATDC5 cells overexpressing ALK2^WT^ or mutant ALK2^R206H^ receptors in regular medium (5% FCS) and serum starvation (starvation) conditions. GFP-positive and RFP-positive cells were detected by fluorescence emitted under 488 nm and 561 nm excitation lasers, respectively. Representative histograms of a single experiment with median fluorescence intensity of GFP and RFP are shown. Cumulative plots of six independent experiments show mean ± SD of GFP/RFP fluorescence ratio in serum starvation conditions versus regular medium (DMEM F12 5% FBS). Statistical analysis: One-way ANOVA (Tukey’s multiple comparison) test was performed (ns = not significant; **P ≤ 0.01; ****P ≤ 0.0001) (**B**) Representative immunoblotting and relative densitometric analysis (*n* = 3) on total protein extracts derived from ATDC5 cells grown in regular medium (5% FBS) or in serum starvation condition (starvation). Tubulin and HSP90 were used as loading control. Statistical analysis: unpaired *t*-test (ns = not significant; **P ≤ 0.01; ****P ≤ 0.0001) (**C**) Confocal quantification of the number of autophagic vesicles in ATDC5 cells in starvation condition after loading with 50 µM MDC. Data analysis of MDC spot number was performed using the Harmony software (ver 4.5) of the Opera Phenix high-content system. Scale bar, 10 μm. Statistical analysis: unpaired *t-*test (****P ≤ 0.0001).

To investigate whether ALK2 may regulate autophagy in FOP, we used mouse chondrogenic progenitor ATDC5 cells stably overexpressing NanoLuc® tagged ALK2^WT^ or ALK2^R206H^ [4, 24], co-expressing the GFP-LC3-RFP-LC3ΔG fluorescence probe, through retroviral infection [25]. This system allows us to measure autophagic activity by flow cytometry, where autophagic inducers will decrease the GFP/RFP ratio, and autophagy blockers will increase the ratio [25]. Upon autophagy induction through serum starvation, the GFP/RFP ratio was reduced by ~40% in ALK2^WT^ cells and only ~10% in ALK2^R206H^ cells, suggesting that ALK2^R206H^ overexpressing chondrogenic progenitor cells are less sensitive to autophagy flux induction when compared to ALK2^WT^ overexpressing cells (Fig. 1A). To verify this data, we studied the protein levels of the autophagic marker p62, a well-known substrate for autophagic degradation [26]. In ATDC5-ALK2^WT^, p62 protein levels were reduced upon serum starvation, while p62 remained higher in ALK2^R206H^ cells (Fig. 1B). We then decided to evaluate the effect of ALK2^R206H^ expression on the formation of autophagic vacuoles. Monodansylcadaverine (MDC) is a specific marker for autolysosomes [27, 28]. MDC accumulates inside autophagosomes, and its fluorescence increases after the fusion of autophagosomes with lysosomes due to the acidic environment. ATDC5 ALK2^WT^ cells and ALK2^R206H^ cells were serum deprived and subsequently loaded with MDC (Fig. 1C). High-content confocal imaging revealed a significant increase in the number of autophagic vacuoles in both cell lines following starvation (Fig. 1C). Nevertheless, compared to control cells, ALK2^R206H^ expressing cells exhibited a significant reduction in the number of autolysosome dots in starvation conditions (Fig. 1C). These results suggest that the autophagic machinery remains functional in both control and mutant cell lines, although overexpression of ALK2^R206H^ partially hampers the autophagic response.

### Autophagy-dependent degradation of ALK2 is partially impaired by the p.R206H mutation

**Fig. 2:**
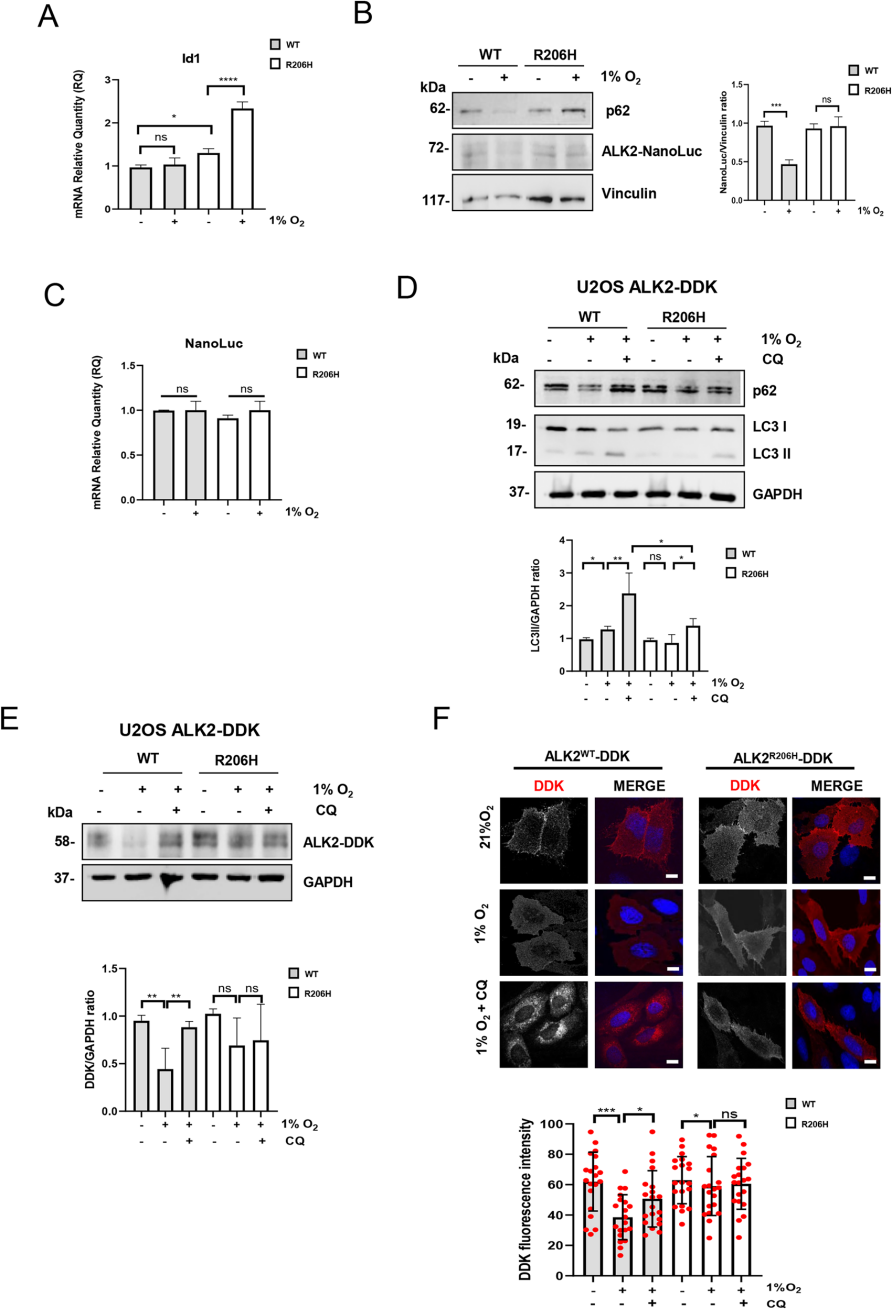
Degradation of ALK2 by autophagy is partially impaired by the p.R206H mutation in hypoxic conditions. **A** Histograms show the mRNA expression for the indicated genes expressed as fold increase (mean ± SD) from three independent experiments in ATDC5 cells overexpressing ALK2^WT^ or mutant ALK2^R206H^ in normoxic (21% O_2_) and hypoxic conditions (1% O_2_,16 h). GAPDH mRNA was used to normalize data. **B** Immunoblotting and corresponding densitometric analysis with the indicated antibodies of ATDC5 cells overexpressing ALK2^WT^ or mutant ALK2^R206H^ in normoxic (21% O_2_) and hypoxic conditions (1% O_2_,16 h) (**C**) Histograms show the mRNA expression for the indicated gene expressed as fold increase (mean ± SD) from three independent experiments in ATDC5 cells overexpressing ALK2^WT^ or mutant ALK2^R206H^ in normoxic (21% O_2_) and hypoxic conditions (1% O_2_,16 h). GAPDH mRNA was used to normalize data. **D**, **E** Immunoblotting and relative densitometric analysis with the indicated antibodies of U2OS cells overexpressing ALK2^WT^ or mutant ALK2^R206H^ DDK tagged, in normoxic (21% O_2_) and hypoxic conditions (1% O_2_,16 h) and treated, as indicated, with 20 μM CQ (2 h). **F** Confocal microscopy analysis using anti-DDK (red) in U2OS cells expressing ALK2^WT^ or mutant ALK2^R206H^ DDK tagged receptors treated or not, as indicated, with chloroquine (CQ, 20 μM, 2 h) in normoxic (21% O_2_) and hypoxic conditions (1% O_2_,16 h). DNA was counterstained with DAPI (blue). Scale bar 10 µm. DDK fluorescence intensity was measured by using Fiji software (ImageJ). At least 50 cells from 3 independent experiments were analyzed unpaired *t*-test (Welch’s correction) was performed. Data are presented as mean ± SD of at least three independent experiments. Symbols (dots) represent individual cells. Statistical analysis: unpaired (ns = not significant; *P ≤ 0.05; **P ≤ 0.01; ***P < 0.001 ****P ≤ 0.0001).

The hypoxic microenvironment is essential in the early events of HO in FOP by amplifying mutant receptor signaling [15, 16]. Importantly, hypoxia is a strong inducer of autophagy [22]. Therefore, we investigated whether hypoxia may influence the impact of ALK2^R206H^ on autophagy. Consistent with previous results [15], the expression of the ALK2-downstream target gene *Id1* and the hypoxic response gene *Hif2α* were induced under hypoxia in ALK2^R206H^ cells, as well as the protein stabilization of HIF-1*α* (Fig. 2A and supplementary Fig. S1A, B). This supports the idea that chondrogenic cells expressing the mutant receptor show an amplified response to hypoxia. Notably, exposing ATDC5 ALK2^WT^ cells to hypoxia (1% O_2_) lowered p62 protein levels compared to normoxic conditions (21% O_2_), suggesting an induction of autophagy (Fig. 2B). Importantly, hypoxic ATDC5 ALK2^R206H^ cells showed similar levels of p62 compared to normoxic cells (Fig. 2B), confirming that ALK2^R206H^ overexpressing cells are less sensitive to autophagy induction. Interestingly, we noticed that overexpressed ALK2^R206H^ protein was more stable in response to hypoxia compared to overexpressed NanoLuc-tagged ALK2^WT^ (Fig. 2B), while mRNA levels remained unchanged (Fig. 2C), further suggesting a differential post-translational regulation in mutant cells. To verify these data, we treated U2OS cells over-expressing DDK-tagged ALK2^WT^ or ALK2^R206H^ receptors cells with chloroquine (CQ), an inhibitor of lysosomal acidification and autophagosome degradation, in hypoxic conditions (Fig. 2D).

**Fig. 3:**
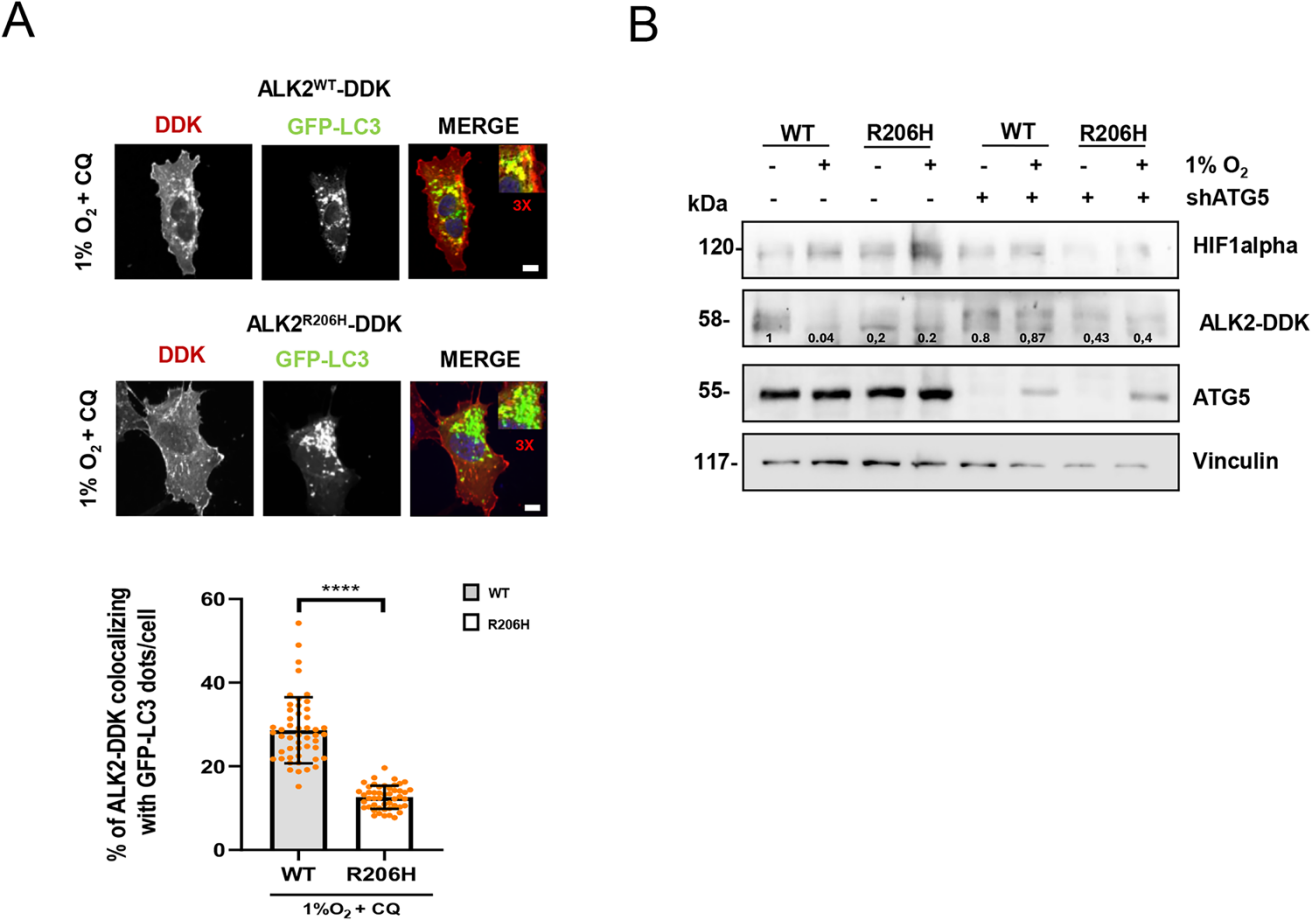
The ALK2 receptor is an autophagy target. **A** Representative images and relative quantification of EGFP-LC3-labeled autophagosomes colocalization with ALK2-DDK receptor. U2OS EGFP-LC3 cells expressing ALK2^WT^ or mutant ALK2^R206H^ DDK tagged receptors were treated with chloroquine (CQ, 20 μM, 2 h) in hypoxic conditions (1% O_2_,16 h). Images were acquired by spinning disk confocal microscopy and analyzed for colocalization of EGFP-LC3 (green) and DDK (red) dots using the ComeDet plugin of the Fiji ImageJ software. Data are presented as mean ± SD and normalized on total EGFP-LC3 dots. DNA was counterstained with DAPI (blue). Insets show a 3-fold enlargement of the boxed areas. Scale bar, 10 μm is shown. At least 50 cells from 3 independent experiments were analyzed unpaired *t*-test (Welch’s correction) was performed. (ns = not significant; *P ≤ 0.05; **P ≤ 0.01; ***P < 0.001 ****P ≤ 0.0001). **B** Immunoblotting and relative densitometric analysis, numbers report the densitometric values of band intensity, with the indicated antibodies of U2OS cells overexpressing ALK2^WT^ or mutant ALK2^R206H^ in normoxic (21% O_2_) and hypoxic conditions (1% O_2_,16 h) and stably infected with lentiviral construct expressing shATG5 RNA interference or expressing an empty vector as control (shCTRL).

In immunoblot experiments, addition of CQ to U2OS ALK2^R206H^ cells in hypoxic conditions (Supplementary figure S1C), did not increase the levels of p62 and slightly increase the level of LC3II, compared to ALK2^WT^ cells, suggesting that the autophagic machinery was functional in the mutant cells but that there was reduced autophagic flux (Fig. 2D and Supplementary Fig. S1D). Moreover, we could show an accumulation of LC3II protein in hypoxic conditions in ALK2^WT^ cells but not in ALK2^R206H^ cells (Fig. 2D). Therefore, we hypothesized that autophagy may be responsible for ALK2^WT^ degradation under hypoxic conditions. To verify this hypothesis, we monitored autophagy-mediated degradation of ALK2 after CQ treatment and hypoxia and we observed an accumulation of ALK2 by CQ only in cells overexpressing the WT receptor (Fig. 2E), Moreover, we examined the sub-cellular distribution of ALK2 in U2OS cells over-expressing DDK-tagged ALK2^WT^ or ALK2^R206H^ receptors by immunofluorescence analysis (Fig. 2F). Strikingly, immunofluorescence detection of ectopic ALK2 in ALK2^WT^ overexpressing cells revealed that this receptor is strongly degraded in hypoxic cells while its levels remain high after CQ treatment (Fig. 2F). Of note, we observed a punctate or dotted staining pattern formation of ALK2^WT^—DDK receptor after CQ treatment (Fig. 2F). To verify that this staining pattern could colocalize with autophagosome structure we expressed EGFP-LC3 in U2OS cells expressing WT and mutant receptor (Fig. 3A). We treated the cells with CQ, to allow accumulation of autophagosome, in hypoxic conditions, and then we evaluated the colocalization of ALK2 DDK tagged receptors with EGFP-LC3 marked autophagosome (Fig. 3A). Immunofluorescence microscopy revealed that 30% of EGFP-LC3 dots colocalize with the ALK2^WT^ receptor, instead 15% of EGFP-LC3 dots colocalize with ALK2^R206H^ receptor in the same conditions (Fig. 3A). This suggest that the p.R206H mutation not only impairs autophagy response but also compromises the degradation of ALK2 by reducing ALK2 association to autophagic vesicles. To further demonstrate that ALK2 is specifically degraded through autophagy, we stably transfected U2OS DDK-tagged ALK2^WT^ or ALK2^R206H^ cells with a short hairpin RNA (shRNA) targeting the essential autophagic gene ATG5 (shATG5) (Fig. 3B). Interestingly, we found that in hypoxic conditions the downregulation of ATG5 impairs the degradation of ALK2^WT^ (Fig. 3B), supporting the idea that ALK2^WT^, but not ALK2^R206H^, is responsive to autophagy-mediated degradation.

### Rapamycin stimulates ALK2^R206H^ degradation through autophagy in FOP cells

**Fig. 4:**
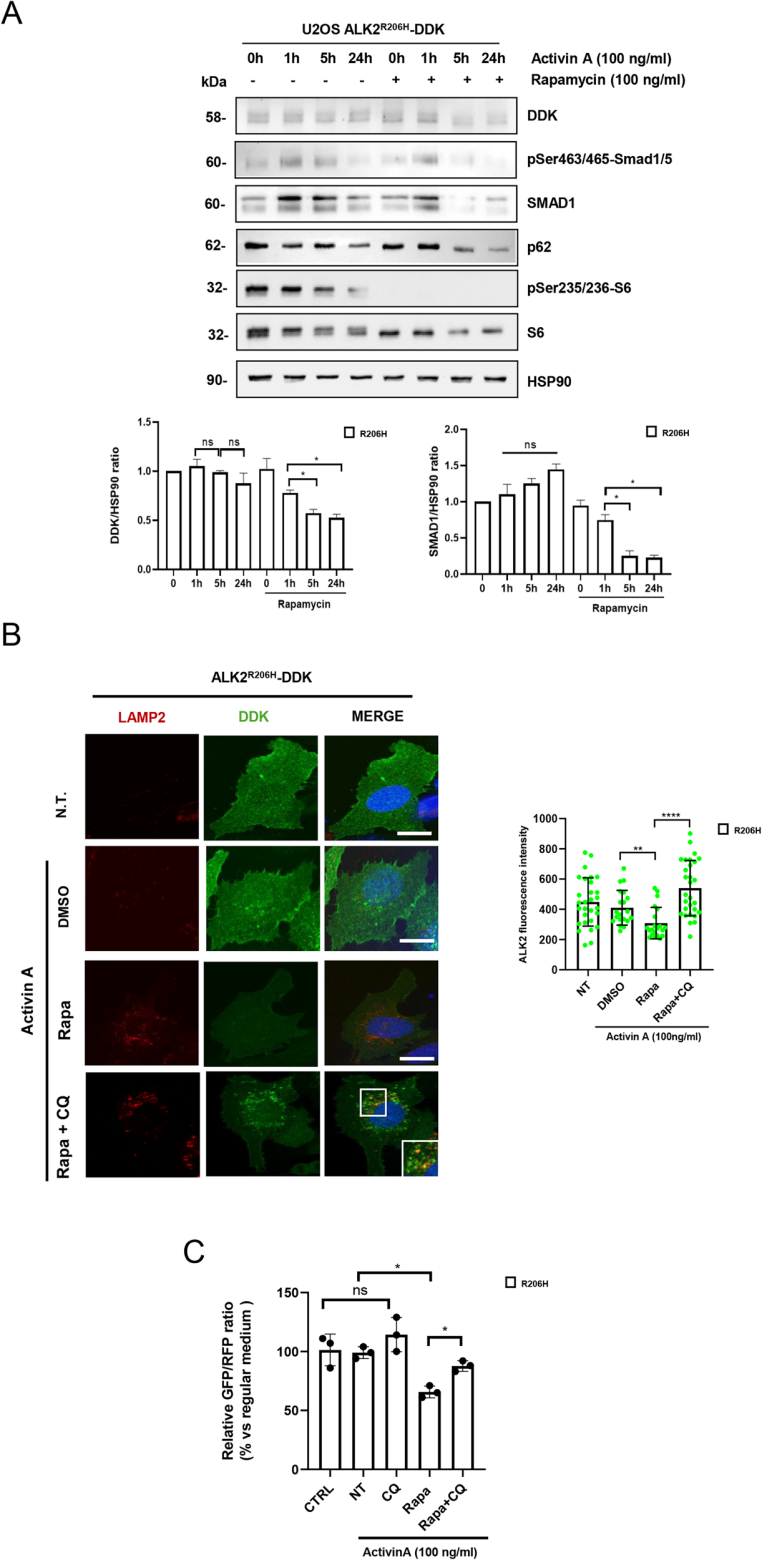
Rapamycin induces ALK2^R206H^ degradation by autophagy upon activin A stimulation. **A** Immunoblotting and relative densitometric analysis with the indicated antibodies of ALK2^R206H^-DDK U2OS cells treated or not, at different time points, with activin A (100 ng/ml) and Rapamycin (Rapa, 100 ng/ml) in serum starvation conditions. Histograms show the mean ± SD of at least three independent experiments. Statistical analysis: unpaired *t*-test (ns = not significant; *P ≤ 0.05; **P ≤ 0.01; ***P ≤ 0.001 ****P ≤ 0.0001). **B** Representative fluorescence images of U2OS cells expressing mutant ALK2^R206H^ DDK tagged receptors treated or not, as indicated, with Rapamycin (Rapa, 100 ng/ml, 5 h) and/or chloroquine (CQ, 20 μM, 2 h), immunostained with anti-LAMP2 (red) and anti-DDK (green) antibodies and counterstained with DNA (DAPI, blue). Scale bar 10 µm. Images were acquired by spinning disk confocal microscopy and analyzed for DDK fluorescence intensity using the Fiji ImageJ software. Data are presented as median with interquartile range. *N* ≥ 20 cells per sample from two independent experiments. Symbols represent individual cells. Insets show a 3-fold enlargement of the boxed areas. Statistical analysis: one-way ANOVA (Tukey’s multiple comparison) test was performed (**P ≤ 0.01; ****P ≤ 0.0001). **C** Flow cytometry analysis of ALK2^R206H^ ATDC5 cells cultured in serum starvation conditions, left untreated (NT) or treated with activin A (100 ng /ml) alone (DMSO), or in combination with Chloroquine (CQ, 20 µM, 2 h), Rapamycin (Rapa, 100 ng/ml, 24 h) for 24 h. Representative histograms show the median fluorescence intensity (MFI) of fluorescence GFP and RFP emitted as a result of excitation with blue (488 nm) and yellow (561 nm) lasers, respectively. Cumulative plots report the mean ± SD of GFP/RFP fluorescence ratio versus non-treated (NT) cells (*n* = 3). Statistical analysis: one-way ANOVA (Tukey’s multiple comparison) test was performed (ns = not significant; **P ≤ 0.01).

Inhibition of the mTOR kinase pathway using rapamycin (Rapa) has been proposed as a promising strategy for the treatment of endochondral ossification triggered by activin A in early hypoxic FOP lesions [23, 29, 30]. Of note, mTOR signaling is a pivotal pathway in regulating hypoxia and autophagy responses [30]. Given that blocking activin signaling via the mutant ALK2^R206H^ receptor has been shown to be an effective therapeutic approach to prevent HO in preclinical FOP models, we next investigated whether autophagy signaling may become a new therapeutic target in FOP. First, we confirmed that the pathogenic activin A signaling described in FOP is recapitulated in our ATDC5 ALK2^R206H^ cells [13]. Indeed, stimulation with recombinant Activin A effectively induced phosphorylation of the ALK2 target SMAD1/5 and the mTOR target ribosomal S6. Consistently, activin A-induced expression of the BMP target gene *Id1* could be measured in ALK2^R206H^ expressing cells (Supplementary Fig. S1E, F). More interestingly, we analyzed whether Rapamycin directly inhibits Activin A signaling in a time-dependent manner, by monitoring phosphorylation of SMAD1/5 in U2OS cells stably expressing DDK-tagged ALK2^R206H^ (Fig. 4A). As expected, activin A was able to induce a robust phosphorylation of SMAD1/5 (Fig. 4A). In the presence of rapamycin, the phosphorylation of SMAD1/5 was only modest and the phosphorylation of the mTOR substrate, ribosomal protein S6 was clearly blocked (Fig. 4A). Interestingly, we observed that after 5 h of co-treatment, ALK2^R206H^-DKK and SMAD1 levels were significantly reduced, when compared to cells treated with activin A or rapamycin separately (Fig. 4A). Of note, the autophagic marker p62 is degraded at the same time point of ALK2^R206H^-DKK protein downregulation (Fig. 4A), thus suggesting that in presence of activin A, mTOR inhibition may induce autophagy-mediated degradation of ALK2^R206H^ in cells expressing the mutant receptor. Accordingly, confocal microscopy revealed that mutant receptor fluorescence signal was strongly decreased in cells exposed concomitantly to activin A and rapamycin (Fig. 4B). Notably, in the presence of CQ an accumulation of the mutant receptor that partially co-localized with the lysosomal marker LAMP2 was evident (Fig. 4B). Indeed, using the GFP-LC3-RFP-LC3ΔG reporter system, we found that Rapamycin co-treatment with activin A rescued the autophagic flux in ALK2^R206H^-expressing ATDC5 cells as shown by ~40% reduction of the GFP/RFP ratio compared to ALK2^R206H^-expressing cells untreated or after activin A alone (Fig. 4C). Consistently, pre-incubation with CQ effectively suppressed this effect (Fig. 4C). Overall, these data suggests that rapamycin-dependent induction of autophagy could impact the degradation of ALK2^R206H^.

**Fig. 5:**
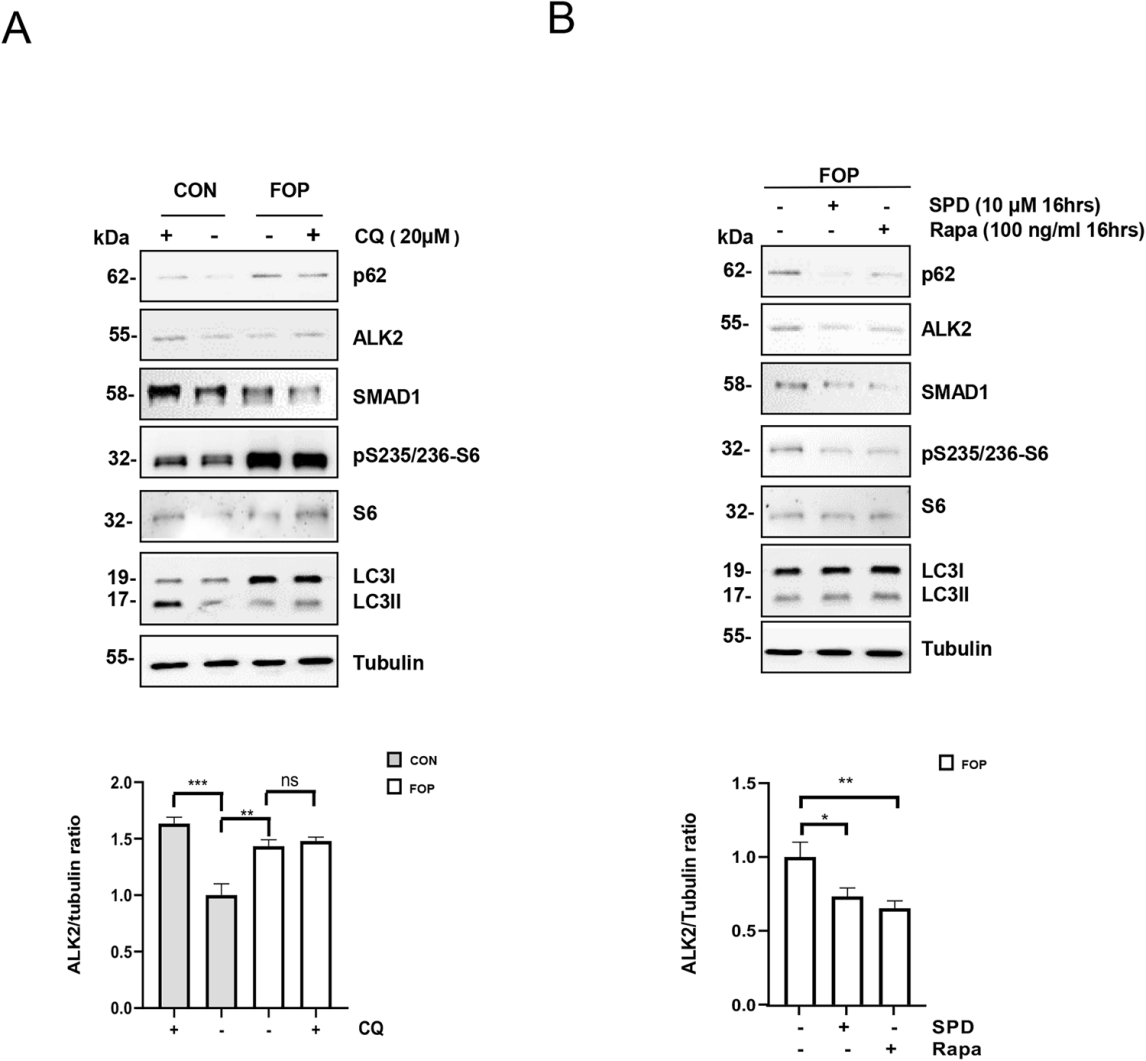
Autophagy is impaired in cells derived from FOP patients, and endogenous ALK2^R206H^ receptor is degraded upon autophagy reactivation. **A** Representative immunoblotting and densitometric analysis with the indicated antibodies of Endothelial colony-forming cells (ECFCs*)* derived from FOP patients (FOP) carrying the R206H mutation or from healthy donors (CON) maintained in serum starvation conditions for 16 h and treated or not with CQ (20 µM, 2 h). **B** Representative immunoblotting and densitometric analysis with the indicated antibodies of total protein extracts from ECFCs cells derived from FOP patient in serum starvation conditions treated or not with Spermidine (SPD, 10 µM) or Rapamycin (Rapa,100 ng/ml) for 16 h. Histograms show the mean ± SD of at least three independent experiments. Statistical analysis: unpaired *t*-test (ns = not significant; *P ≤ 0.05; **P ≤ 0.01; ***P < 0.001 ****P ≤ 0.0001).

To verify our findings in a disease-relevant cell type, we made use of primary Endothelial colony forming cells (ECFCs) derived from control (CON) and FOP donors (FOP) [31]. These cells have been shown to recapitulate the molecular mechanisms underlying FOP, including the neo-functional response to activin A [31]. Upon serum starvation, only FOP cells did not show increased levels of p62 in response to CQ (Fig. 5A), supporting the hypothesis that the autophagic flux is impaired in FOP cells compared to control cells. Although basal LC3II levels are different in FOP cells compared to control cells, we were able to detect an accumulation of LC3II upon CQ treatment only in cells derived from healthy donors (Fig. 5A). Of note, we observed that ALK2 and SMAD1 proteins level accumulated in control cells but not in FOP cells after CQ treatment (Fig. 5A). According to the literature, also mTOR kinase activity was elevated in FOP cells, compared to control cells (Fig. 5A). Then, we decided to reactivate autophagy in FOP cells using Rapamycin or Spermidine (SPD) for 16 h (Fig. 5B). SPD is a well-known autophagy inducer [32] (Supplementary Fig. S2A). Interestingly, we could show through p62 degradation and LC3II accumulation that autophagy is reactivated in these cells, which correlates with the inhibition of mTOR kinase activity and with a significant reduction of ALK2 and SMAD1 protein levels in FOP cells treated with Rapa or SPD compared to control cells (Fig. 5B).

Taken together, these results indicate that pharmacological modulation of autophagy may be an approach for the downregulation of aberrant ALK2^R206H^ receptor signaling in FOP.

### Autophagy reactivation may be a promising approach to inhibit Activin-induced chondrogenesis in FOP

**Fig. 6:**
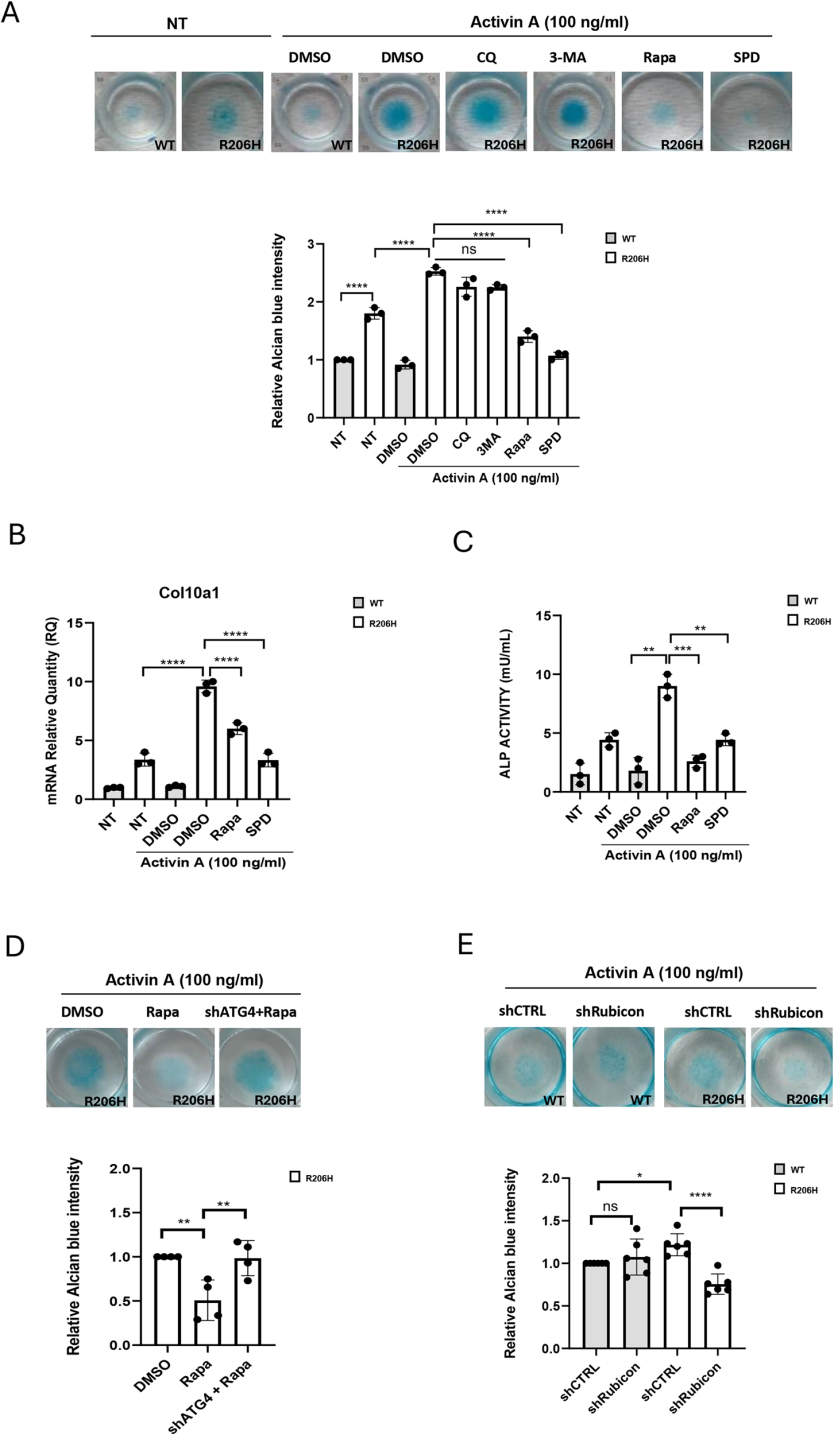
Autophagy reactivation inhibits Activin-induced chondrogenesis in FOP cells. **A** Chondrogenic differentiation micromass assay in ATDC5 ALK2^R206H^ cells and in ATDC5 ALK2^WT^ cells. Cells were incubated for 21 days in differentiation medium containing or not (NT), activin A (100 ng/ml) and treated, as indicated, with autophagy inhibitors Chloroquine (CQ, 20 μM), 3-Methyladenine (3-MA, 20 μM) or autophagy inducers Rapamycin (Rapa, 100 ng/ml), Spermidine (SPD, 20 μM) or DMSO. Representative images of Alcian blue staining. Histograms representing Alcian blue quantification measured by absorbance at 595 nm after solubilization with guanidine hydrochloride. All results represented the mean ± SD of at least three independent experiments. Statistical analysis: One-way ANOVA (Tukey’s multiple comparison) test was performed (**P ≤ 0.01; ***P < 0.001 ****P ≤ 0.0001). **B** Real-time PCR of *Col10a1* mRNA in ATDC5 cells treated or not with activin A for 21 days in micromass cultures. mRplp0 was used to normalize data. All results represented the mean ± SD of at least three independent experiments. Statistical analysis: unpaired *t-*test (**P ≤ 0.01; ***P < 0.001 ****P ≤ 0.0001). **C** Histogram showing measurement of ALP activity in ATDC5 cells treated or not as indicated for 5 days in micromass culture. 5 × 10^4^ cells were homogenized in 1 ml of assay Buffer, diluted 1:10 in assay Buffer, and 80 μl was used to measure ALP activity. Assays were performed following the fluorimetric kit protocol (MAK411, Sigma–Aldrich) and measured at O.D. 405 nm reading with Varioskan LUX Plate Reader. All results represented the mean ± SD of at least three independent experiments. Statistical analysis: A one-way ANOVA (Tukey’s multiple comparison) test was performed (**P ≤ 0.01; ***P < 0.001). Chondrogenic differentiation micromass assay in ATDC5 ALK2^R206H^ cells stable expressing shRNA for ATG4 (shATG4) (**D**) or ATDC5 ALK2^WT^ and ALK2^R206H^ cells stable expressing shRNA for Rubicon (shRubicon) (**E**), upon lentiviral infection. A lentiviral empty vector was used as control (shCTRL). Cells were incubated for 21 days in differentiation medium containing or not (NT), activin A (100 ng/ml) and treated as indicated with Rapamycin (Rapa 100 ng/ml) or DMSO. Representative images of Alcian blue staining and relative histograms showing Alcian blue quantification measured by absorbance at 595 nm after solubilization with guanidine hydrochloride. All results represented the mean ± SD of at least three independent experiments for (**D**) and from six independent experiments for (**E**). Statistical analysis: One-way ANOVA (Tukey’s multiple comparison) test was performed (*P ≤ 0.05; **P ≤ 0.01, ****P ≤ 0.0001).

To support the hypothesis that autophagy reactivation could be a general strategy for targeting Activin-induced chondrogenesis due to pathogenic ALK2^R206H^ signaling, we evaluated whether well-known autophagy regulators may be able to prevent chondrogenic differentiation in ATDC5 cells stably expressing ALK2^WT^ or ALK2^R206H^. Micromass cultures were incubated in chondrogenic medium for 21 days (an established cell model of chondrogenic differentiation) [31, 32], in the presence of recombinant activin A and different autophagic modulators as indicated (Fig. 6A). As expected, autophagy reactivation using rapamycin or SPD effectively inhibited chondrogenic differentiation as assessed through proteoglycan staining with Alcian blue, *Col10a1* expression and ALP activity (Fig. 6A–C). Of note, the autophagic inhibitors CQ and 3-Methyladenine (3-MA) did not affect ALK2^R206H^ dependent chondrogenesis (Fig. 6A–C). To confirm that rapamycin prevents chondrogenic differentiation via autophagy activation, we used RNA interference to knock down the essential autophagy gene *ATG4* (Supplementary Fig. S2B), which rescued chondrogenic differentiation of ALK2^R206H^-expressing cells despite the presence of rapamycin (Fig. 6D). Because rapamycin and spermidine do not selectively affect autophagy, we verified whether autophagy-specific activation can inhibit chondrogenic differentiation. We downregulated Rubicon (Run domain Beclin-1 interacting and cysteine-rich containing) expression in ATDC5 cells (Supplementary Fig. S2C, D) because Rubicon is a potent direct negative regulator of autophagy and a potential target for autophagy inducers [33]. Interestingly, we observed that the downregulation of Rubicon impairs chondrogenic differentiation only in cells expressing the mutant ALK2^R206H^. This further suggests that autophagy activation can inhibit ALK2^R206H^-induced chondrogenic differentiation (Fig. 6E). In conclusion, we identified an interplay between ALK2^R206H^ signaling and autophagy as an important regulator of pathogenic ALK2^R206H^-mediated chondrogenic differentiation in FOP.

## Discussion

In this manuscript, we describe for the first time that the expression of the mutant BMP receptor ALK2^R206H^ impairs the autophagy flux. Interestingly, we discovered that the ALK2 receptor levels are regulated through autophagy, while ALK2^R206H^ autophagy-mediated degradation is partially impaired. To investigate a potential therapeutic translation of these findings, we examined whether autophagy inducers like rapamycin and spermidine could restore ALK2 signaling during chondrogenesis in ALK2^R206H^ expressing ATDC5 and FOP patient-derived cells.

Mechanistically, we have demonstrated that a reduced autophagic flux induced by the mutant ALK2^R206H^ can underlie ALK2 protein stabilization. This finding is further supported by the reduced colocalization between ALK2^R206H^ and the autophagosome marker EGFP-LC3, compared to ALK2^WT^, in response to CQ and hypoxia. In line with these results, the induction of autophagy with rapamycin also led to a decrease in ALK2^R206H^ protein levels (both endogenous and exogenous) in a time-dependent manner, following stimulation with activin A. Finally, reactivation of autophagy with rapamycin upon activin A stimulation increased the lysosomal localization of ALK2^R206H^, suggesting that reactivation of autophagy in FOP cells could lead to lysosomal degradation of the mutant receptor Given that hypoxia may prolong mutant ALK2 signaling by receptor retention [15], we hypothesized that autophagy is the process regulating the timing of ALK2 endosomal retention. Most transmembrane proteins are not degraded via autophagy but through endosomal sorting complex required for transport (ESCRT) following endocytosis. However, transmembrane receptors within the TGF-β superfamily, like TGFβR [34] and BMPR2 [35], are degraded in an autophagy-dependent manner through a close interaction between endosomes and autophagy signaling [36]. We hypothesize that the alteration of autophagy in FOP may impair the ESCRT machinery, thus indirectly regulating the retention of ALK2 in the endosome. Alternatively, ALK2 could be directly recruited to the autophagosome through direct interaction with selective autophagosome proteins, like LC3C [37]. The p.R206H mutation may compromise this interaction, causing a decreased degradation compared to wild-type controls. Of note, we found that autophagy also regulates SMAD1 protein trafficking. This is in line with recent studies suggesting that autophagy could directly impact SMAD1 nuclear localization [34], therefore regulating ALK2-SMAD1 signaling at multiple levels. Future studies should focus on uncovering the molecular mechanisms of autophagy-dependent regulation of mutant ALK2 trafficking, including the identification of adaptor proteins mediating endosome-autophagosome recycling, ALK2/SMAD1 degradation, specific ubiquitin ligases, and post-translational modifications involved in this process.

Our findings may open new potential therapeutic approaches for FOP. As aforementioned, rapamycin, a commercially available and commonly used mTOR inhibitor, showed potent therapeutic effects on activin-induced HO in different in vivo models triggered by activin A, suggesting a hyperactivation of mTOR downstream of ALK2^R206H^ [38]. Here, we demonstrated for the first time that ALK2^R206H^ expression compromises autophagy induction, suggesting that autophagy modulation could be the main driving mechanism of rapamycin’s therapeutic effect. Under conditions mimicking in vivo chondrogenic differentiation (i.e. hypoxia), we showed a reduced autophagic flux in cells expressing ALK2^R206H^. Consistently, Yang et al. reported reduced autophagic flux in a mouse model where constitutively active ACVR1/ALK2 drove cranial neural crest cells toward an aberrant chondrogenic fate, highlighting a role for BMP signaling in mTOR activation [39]. Therefore, we hypothesized that mTOR could be driving the effects of mutant ALK2 signaling on autophagy. Of note, we demonstrated that rapamycin was able to reactivate autophagy in ALK2^R206H^ overexpressing ATDC5 and FOP patient-derived cells, suggesting that the previously described therapeutic effect of rapamycin in FOP could be mediated by autophagy. Since untenable side effects have been associated with rapamycin for its potential application in FOP [40], we evaluated a different autophagic activator with less documented side effects: the natural drug Spermidine (SPD) [41]. Spermidine-dependent reactivation of autophagy is known to be mediated by the inhibition of mTOR activity via AMPK activation and p300 inhibition [42–44]. Similarly to Rapamycin, SPD could reduce activin A-induced chondrogenic differentiation in ALK2^R206H^ cells. To discard that the autophagic-specific activation induced by rapamycin and spermidine is responsible for chondrogenesis inhibition, we genetically knocked down the autophagic inhibitor Rubicon to reactivate autophagy. Consistent with our previous results, we found that the absence of Rubicon inhibits chondrogenic differentiation driven by ALK2^R206H^. These data could not exclude that additional pathways, regulated by rapamycin and spermidine, could contribute to the inhibition of chondrogenic differentiation.

**Fig. 7:**
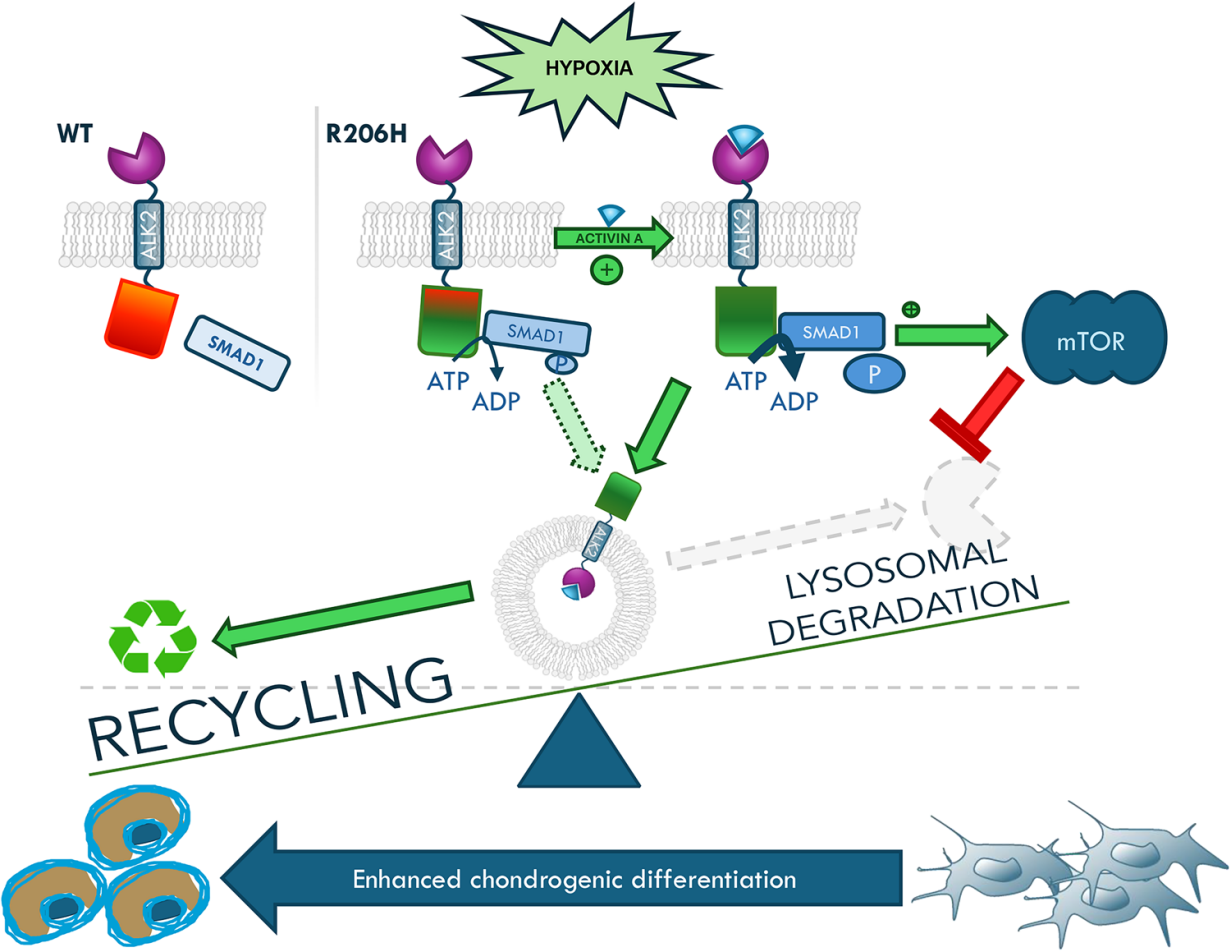
Schematic illustration showing a simplified model of the interplay between the mutant ALK2^R206H^ receptor (without showing the type I/type II receptors tetramer) and the mTOR/autophagy signaling in the regulation of the balance between receptor degradation and chondrogenesis upon hypoxic conditions or activin A stimulation.

Overall, our data demonstrate that the induction of autophagic flux is partially impaired in ALK2^R206H^ expressing cells, and that the treatment of FOP cells with autophagic inducers could have potential therapeutic efficacy blocking HO (Fig. 7). Altogether, this study sheds light on the importance of autophagy dysregulation in FOP pathology and highlights unexplored molecular mechanisms underlying ALK2 receptor signaling regulation.

## Materials and methods

### Cell culture

Mouse teratocarcinoma ATDC5 cell lines stably expressing ALK2-WT-nanoluc and ALK2-R206H-nanoluc (NanoLuc® vector, Promega), described in [4], were cultured in DMEM-F12 (1:1) medium supplemented with 5%FBS (Sigma–Aldrich), streptomycin 10 mg/ml, penicillin 10,000 U/ml (Sigma–Aldrich) at 37 °C and 5% CO_2_, and routinely tested for mycoplasma contamination. ATDC5 cell lines stably expressing GFP-LC3-RFP-LC3ΔG probe were generated through retroviral infection. Retroviral particles were obtained by co-transfection of HEK293T cells with pGAG and VSVG packaging vectors and the plasmid pMRX-IP-GFP-LC3-RFP-LC3ΔG (Addgene plasmid # 84572). Stable transgenic ATDC5 cell lines expressing shRNA for ATG4 or shRubicon, were generated by infection with the lentiviral vectors from: Vector Builder: pLV[shRNA]-Puro-U6>mAtg4b[shRNA#1] VB900148-6043fsk, pLV[shRNA]-Puro-U6>mRubcn[shRNA#1] (VB900168-4673rbt) and pLV[shRNA]-Puro as control (shCTRL). U2OS cells were grown in DMEM (Sigma–Aldrich), supplemented with 10% FBS streptomycin 10 mg/ml, penicillin 10,000 U/ml (Sigma–Aldrich) at 37 °C and 5% CO_2_, and routinely tested for mycoplasma contamination. Stable cell lines expressing pCVM6-ALK2-MycDDK (Origene RC207486R) and pCVM6-ALK2-R206H- MycDDK (Origene RC402551) were generated by lipofectamine 2000 (Life Technologies) transfection and G418 (500 µg/ml, Sigma–Aldrich) selection. Stable transgenic U2OS cell lines expressing shRNA for ATG5 were generated by infection with the lentiviral vectors from Vector Builder: pLV[shRNA]-Puro-U6 > hATG5[shRNA#1] (VB900148-6040xna) or pLV[shRNA]-Puro as control (shCTRL). All stable cell lines were generated by lentivirus-mediated expression using lentivirus produced in HEK293T cells by co-transfecting lentiviral vectors (described above) together with respective plasmids encoding for gag-pol (PAX2) and VSV-G (pMD2.G) proteins. The viral supernatant was collected 48 h post-transfection, filtered through a 0.45 µm pore size filter, and added to the cells in the presence of 2 *µ*g/ml polybrene. For hypoxia induction, the cells were grown in a hypoxic cabin (1% O_2_; HypoxicLab^TM^, oXFORD oPTRONIX) in the Hyp-ACB facility (Sapienza University of Roma) for 16 h where hypoxia was sustained during experimental treatments. Human endothelial colony-forming cells (ECFCs) are cell lines described in [31].

### Immunoblotting

Protein extraction through cell lysis was performed at 4 °C. Cell pellets were resuspended using RIPA buffer (25 mM Tris pH 7.5, 150 mM NaCl, 0.1% SDS, 1% Sodium Deoxycholate, 1% NP40) supplemented with protease and phosphatase inhibitors: cocktail of protease inhibitors (Leupeptin 1 mg/ml, Aprotinin 1 mg/ml, Pepstatin 10 mg/ml), 1 mM PMSF, 25 mM NAF, 1 mM Na3VO4 (Orthovanadate sodium), 10 mg/ml TPCK (Tosyl 79 phenylalanyl chloromethyl ketone), 40 mM β-glycerophosphate. The resuspended pellets were incubated for 10 min on ice and centrifuged at 12.000 rpm for 10 min at 4 °C. Supernatants were recovered, and protein concentration was determined using the Bradford assay. For immunoblotting, 20–30 μg of protein extract were separated by sodium dodecyl sulfate-polyacrylamide gel electrophoresis (SDS-PAGE), blotted on nitrocellulose membrane, and stained with the specific primary antibodies: anti-Phospho-S6 Ribosomal Protein (Ser235/236) (D57.2.2E,Cell Signaling Technology), anti-p62 (SQSTM1) (PM045; MBL International), anti-GAPDH (D16H11) (Cell Signaling Technology), anti-LC3B (Sigma–Aldrich, L7543), anti-NanoLuc (Promega Corporation, N700A), anti-DDK (FLAG) (Origene, TA50011-100), anti ALK2/ACVR1 (SinoBiological. 10227-T24), anti-SMAD1 (Cell Signaling Technology, 9743S), anti-phospho-Smad 1/5 (Ser463/465) (Cell Signaling Technology, D5B10), anti-phosho-Smad2 (Ser465/467)/Smad3 (Ser423/425) (Cell Signaling Technology, D27F4), anti-ATG5 (Proteinech, 10181-2-AP), anti-Rubicon (Proteinech 21444-1-AP),anti-tubulin (Sigma–Aldrich, 1:3000) and anti-Hsp90 (F8, Santa Cruz Biotechnology, 1/2000), anti-Vinculin (Santa-Cruz, SC-73614)Secondary antibodies were anti-mouse (Bio-Rad Laboratories, 1706515) or anti-rabbit (Bio-Rad Laboratories, 1706516) IgG-horseradish peroxidase-conjugated antibodies (Cell Signaling), incubated at a dilution of 1:4000. All immunoblots were imaged upon enhanced chemiluminescence (ECL, Sigma–Aldrich) activation. Densitometric evaluation of band intensity was performed using Fiji-ImageJ software, and values were normalized to loading controls. All original Western blots are presented in Supplementary Fig. S3.

### Immunofluorescence

Cells were seeded on coverslips and grown at 37 °C and 5% CO_2._ Detection of autophagosomal structures was performed by fluorescence microscopy observing LC3B puncta in EGFP-LC3B-U2OS expressing cells, kindly provided by Dr.Daniela Trisciuoglio (IBPM, CNR, Rome) [45] After treatments, the growth medium was removed, and cells were washed with PBS and fixed in 3.7% formaldehyde in PBS for 10 min at RT. After washing, cells were permeabilized with a PBS/0.2%Triton X-100 solution for 5 min, blocked with 3% BSA in 0.1% Triton X-100 in PBS for 1 h at RT and incubated overnight with primary antibodies (DDK, 1:200, clone OTI4C5; Origene,; LAMP2: 1:1:200, sc-18822 Santa Cruz Biotechnology) in a humid chamber at 4 °C. Secondary antibodies,anti-rabbit Cy3 (Jackson ImmunoResearch, 711,165,152, 1:200) and anti-mouse Rhodamine Red-X (Jackson ImmunoResearch, 715,295,150, 1:400) were incubated for 1 h at RT and cells were counterstained with 4,6-diamidino-2-phenylindole (DAPI, 0.1 μg/ml; Sigma–Aldrich) and mounted using the Fluoromount Aqueous Mounting Medium (Sigma–Aldrich). Images were acquired by confocal microscopy (ZEISS LSM800) and analyzed by using Fiji-ImageJ software; quantification of particles colocalizing in two channels was performed by using the plugin ComDet (0.5.2).

### Micromass assay

ATDC5 cells were trypsinized and washed with PBS. 3 × 10^5^ cells were resuspended in 20 μL of culture medium. Gently, a 20 μL drop was seeded in the center of each well in a 24-well plate and incubated in the incubator for 3 h. Then, 500 μL of DMEM-F12/5% FBS medium containing 1X ITS (Insulin-transferrin-sodium selenite 100X, Sigma–Aldrich, I3146) was carefully added to the wells. After 24 h the medium was replaced by DMEM-F12/5% FBS medium containing 1X ITS supplemented with activin A (100 ng/mL, Sigma–Aldrich, A494) with or without Chloroquine (CQ, 20 μM Sigma–Aldrich, C6628), Rapamycin (Rapa, 100 ng/ml, Sigma–Aldrich, R0395), 3-methyladenine (3-MA, 20 μM Sigma–Aldrich, M9281) or Spermidine (SPD, 20 μM Sigma–Aldrich, S0266). Cultures grew for 14 days, refreshing the medium every 3–4 days. Afterward, cells were fixed for 15 min in 500 μL of 10% formaldehyde in PBS. Then, the fixative solution was removed, and cells were incubated overnight at 37 °C in Alcian Blue staining solution (Sigma–Aldrich, B8438). After cell washing, the staining was solubilized in 250 μL of 6 M guanidine hydrochloride, and quantification was performed by measuring absorbance at 595 nm.

### RT-qPCR

Cells were homogenized in TRI Reagent (Thermo Fisher), and RNA was extracted using Direct-zol RNA Miniprep plus kit (Zymo Research) following the manufacturer’s protocol. 500 ng of total RNA was used for cDNA synthesis using the SensiFAST™ cDNA Synthesis Kit (Bioline). One Microliter of cDNA was employed to quantify the transcripts by RT-PCR using SYBR Green mix (SensiFAST SYBR HIROX Mix, Meridian Bioscience) and gene-specific primers on a QuantStudio™ 5 Real-Time PCR System (ThermoFisher Scientific). Changes in mRNA levels were determined relative to mGAPDH or mRplp0 housekeeping controls.

The following primers were used:

mRubicon For 5′ GGTTCTAAGCTCACCAGCCAT 3′

mRubicon Rev 5′ CCAGTGCTCCCTCCTGCT 3′

mATG4B For 5′ CCAGCTATTGATTGGAGGTGGA 3′

mATG4B Rev 5′- CCAACTCCCATTTGCGCTATC 3′

mId1 For 5′ CGAGGTGAGGCGGCAT 3′

mId1 Rev 5′ GAGTCCATCTGGTCCCTCAG 3′

mCol10a1 For 5′ CATCTCCCAGCACCAGAATC 3′

mCol10a1 Rev 5′ GCTAGCAAGTGGGCCCTTTA 3′

mHif2α For 5′ TGAGTTGGCTCATGAGTTGC 3′

mHif2α Rev 5′ CTCACGGATCTCCTCATGGT 3′

NanoLuc For 5′ GGTGTCCGTAACTCCGATCC 3′

NanoLuc Rev 5′ TGCCATAGTGCAGGATCACC 3′

mGAPDH For 5′ ACCCTTAAGAGGGATGCTGC 3′

mGAPDH Rev 5′ CCCAATACGGCCAAATCCGT 3′

mRplp0 For 5′ GCAGGTGTTTGACAACGGCAG 3′

mRplp0 Rev 5′ GATGATGGAGTGTGGCACCGA 3′

### Monodansylcadaverine (MDC) assay

This assay is based on the autofluorescent compound monodansylcadaverine (MDC) for in vivo labeling of autophagic vacuoles and has been carried out as previously described [27, 46]. Briefly, 1.5 × 104 ATDC5^ALK2WT^ and ATDC5^ALK2R206H^ cells seeded in 96-well plates and cultured in different experimental conditions were loaded with 50 μM MDC (Sigma–Aldrich) in culture medium and incubated at 37 °C for 10 min. The cells were then washed with fresh culture medium without MDC and left in PBS for analysis. High-content imaging and data analysis were performed using the Opera Phenix high-content screening system (PerkinElmer). Wells were analyzed in confocal mode, using a 40X water-immersion objective. MDC was excited at 405 nm, and its emission measured between 435 and 550 nm. Data analysis of MDC signal spots was performed using the Harmony software (version 4.9) of the Opera Phenix high-content system. For each field, the applied algorithm quantified the number of fluorescence spots and the cell area for normalization [46].

### Alkaline phosphatase assay

ATDC5 cells were grown for 21 days with or without a differentiation medium (DMEM-F12 5% FBS containing ITS and Activin A 100 ng/ml). Cells were harvested, and 5 × 10^4^ cells were homogenized in 1 ml of assay buffer, diluted 1:10 in assay buffer, and 80 μl were used to measure alkaline phosphate (ALP) activity using Alkaline Phosphatase Activity Fluorometric Assay Kit (MAK411-1KT, Sigma–Aldrich). Assays were performed according to the manufacturer’s protocol, and OD was measured at 405 nm with Varioskan LUX Plate Reader as described in [23].

### FACS analysis

Cells were washed with PBS and detached with Trypsin-ETDA 0.05% at 37 °C for 1 min, being careful not to prolong trypsinization time. Then, cells were resuspended in 1 ml of growth medium and immediately put on ice, centrifuged at 800 × *g* for 3 min at 4 °C, and the pellets resuspended in cold PBS. Samples were acquired by using a CytoFLEX LX (Beckman Coulter) and analyzed using FlowJo-10 software (10.3.0). Histograms of fluorescence intensity versus cell count and the GFP/RFP fluorescence ratio (as a percentage of the mean of cells cultured in regular medium) are shown. Data represent mean ± SEM.

### Statistical analysis

Data from at least three independent biological replicates (represented by solid dots in the graphs) are annotated as mean ± standard deviation (S.D.). The sample size, indicated in figure legends, was not pre-determined. No blinded analysis was performed. All statistical tests indicated in the respective figure legends were performed with GraphPad Prism 8.0.2 software (San Diego, California, USA). The unpaired *t*-test and the ordinary one-way ANOVA multiple comparison test were used for measurements of continuous variables, depending on whether the samples were normally or non-normally distributed (assessed by the Shapiro–Wilk normality test). Statistical significance (*) was set at *p* < 0.05.

## Supplementary information

Supplementary Figure S1

Supplementary Figure S2

Suplementary Figures legends

Original data

## Data Availability

Uncropped original western blots used in this manuscript can be found in “Supplemental Material.“ All data generated during this study and supporting the present results are available from the corresponding author upon reasonable request.
