# Supplementary figures and images for: Interplay between ALK2R206H mutant receptor and autophagy signaling regulates receptor stability and its chondrogenic functions
Source: Cell Death Discov. 2025 Mar 22;11:117. doi: 10.1038/s41420-025-02393-0 (PMC11929866; doi:10.1038/s41420-025-02393-0)

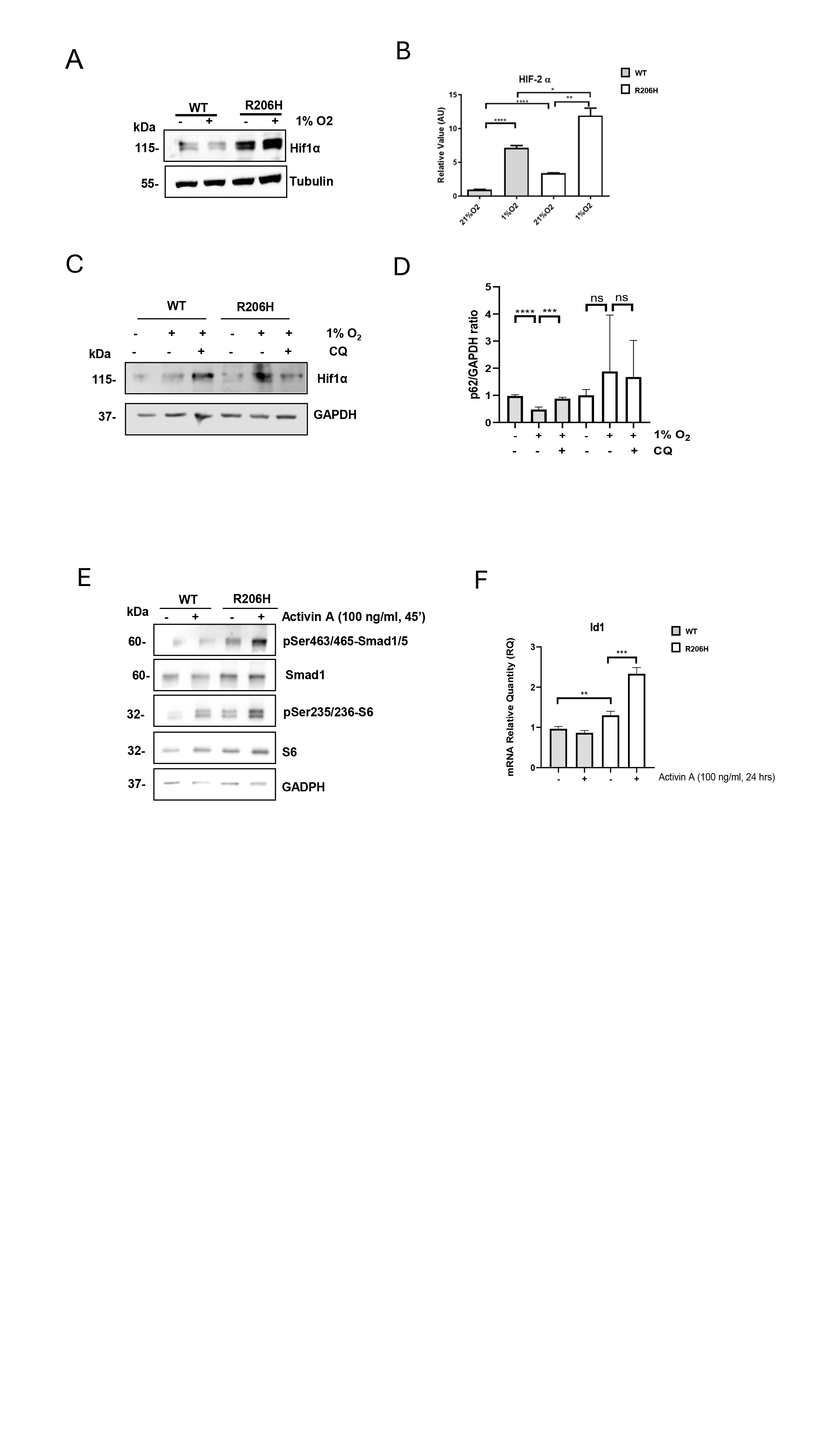

Supplement: Supplementary file 1 — Supplementary Figure S1 [file 41420_2025_2393_MOESM1_ESM.tif]

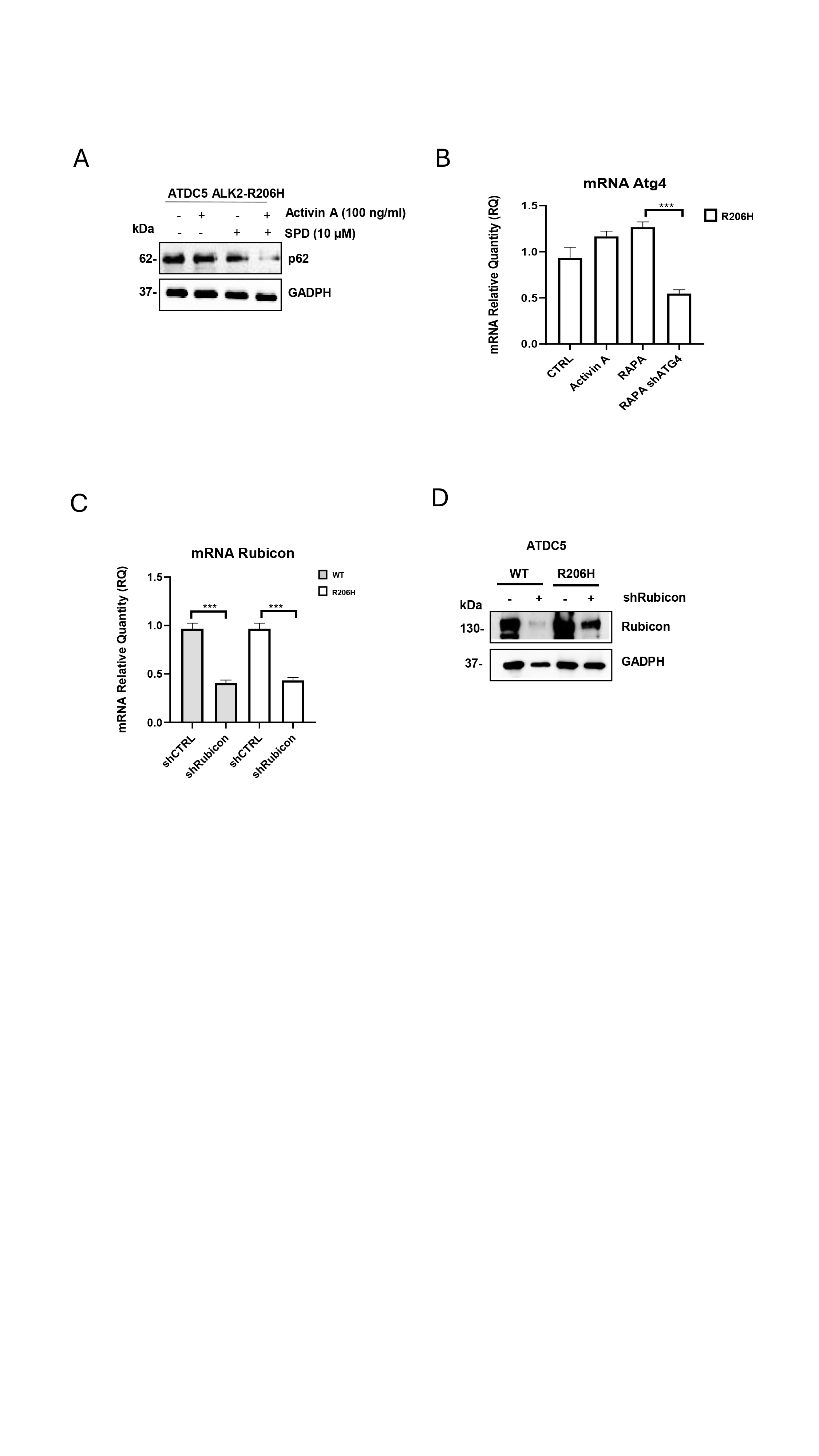

Supplement: Supplementary file 2 — Supplementary Figure S2 [file 41420_2025_2393_MOESM2_ESM.tif]
