# Supplementary material for: Interplay between ALK2R206H mutant receptor and autophagy signaling regulates receptor stability and its chondrogenic functions
Source: Cell Death Discov. 2025 Mar 22;11:117. doi: 10.1038/s41420-025-02393-0 (PMC11929866; doi:10.1038/s41420-025-02393-0)
Supplement: Supplementary file 3 — Suplementary Figures legends [file 41420_2025_2393_MOESM3_ESM.docx]

**Supplementary Figure S1**. **(A)** Representative immunoblot for HIF-1α with the indicated antibodies in ATDC5 ALK2 ^WT^ and ALK2 ^R206H^ cells treated or not, as indicated, in hypoxic conditions (1% O_2_) **(B)** Histograms show the average mRNA expression for *Hif2α* mRNA (mean ± SD); fold increases are from three independent experiments; GAPDH was used to normalize data) **(C)** Representative immunoblot for HIF-1α with the indicated antibodies in U2OS ALK2 ^WT^ and ALK2 ^R206H^ cells treated or not, as indicated, in hypoxic conditions (1% O_2_) in the presence or not of CQ **(D)** Quantification of results presented in Figure 2D, obtained by the densitometric analysis of 2-4 immunoblots. Values were normalized to GAPDH as loading control and are reported as fold change (FC) over the control value. *P<0.05; **P<0.01; ****P<0.0001 **(E)** Immunoblotting and relative densitometric analysis of total protein extract with the indicated antibodies in ATDC5 cells treated or not, as indicated, with activin A (100 ng/ml) **(F)** Real-time PCR for *mID1* mRNA in ATDC5 ALK2 ^R206H^ cells treated or not with activin A as indicated. Statistical analysis: One-way ANOVA (Tukey’s multiple comparison) test was performed (*P≤0.05; **P≤0.01; ; ****P* < 0.001 ****P≤0.0001)

**Supplementary Figure S2. (A)** Representatives immunoblot for p62 on ATDC5 ALK2 ^R206H^ cells treated or not with activin A (100 ng/ml) or Spermidine (SPD, 20 μM) for 16 hrs **(B) (C)** Real-time PCR for *ATG4* mRNA **(B)** and Rubicon mRNA **(C)** in ATDC5 ALK2^WT^ and ALK2 ^R206H^ cells treated or not with activin A or Rapamycin as indicated in Figure 6D and 6E.**(D)** Immunoblotting and relative densitometric analysis of total protein extract with the indicated antibodies in ATDC5 cells . Statistical analysis: One-way ANOVA (Tukey’s multiple comparison) test was performed (*P≤0.05; **P≤0.01; ; ****P* < 0.001 ****P≤0.0001)
