# Supplementary material for: Interplay between ALK2R206H mutant receptor and autophagy signaling regulates receptor stability and its chondrogenic functions
Source: Cell Death Discov. 2025 Mar 22;11:117. doi: 10.1038/s41420-025-02393-0 (PMC11929866; doi:10.1038/s41420-025-02393-0)

Figure 1C

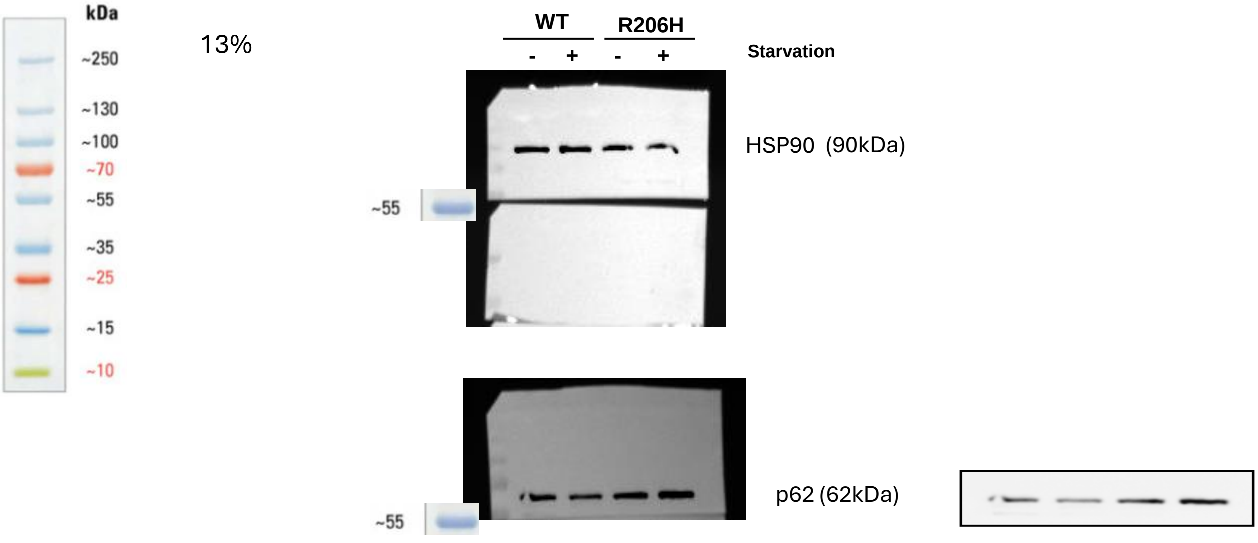

Figure 2B

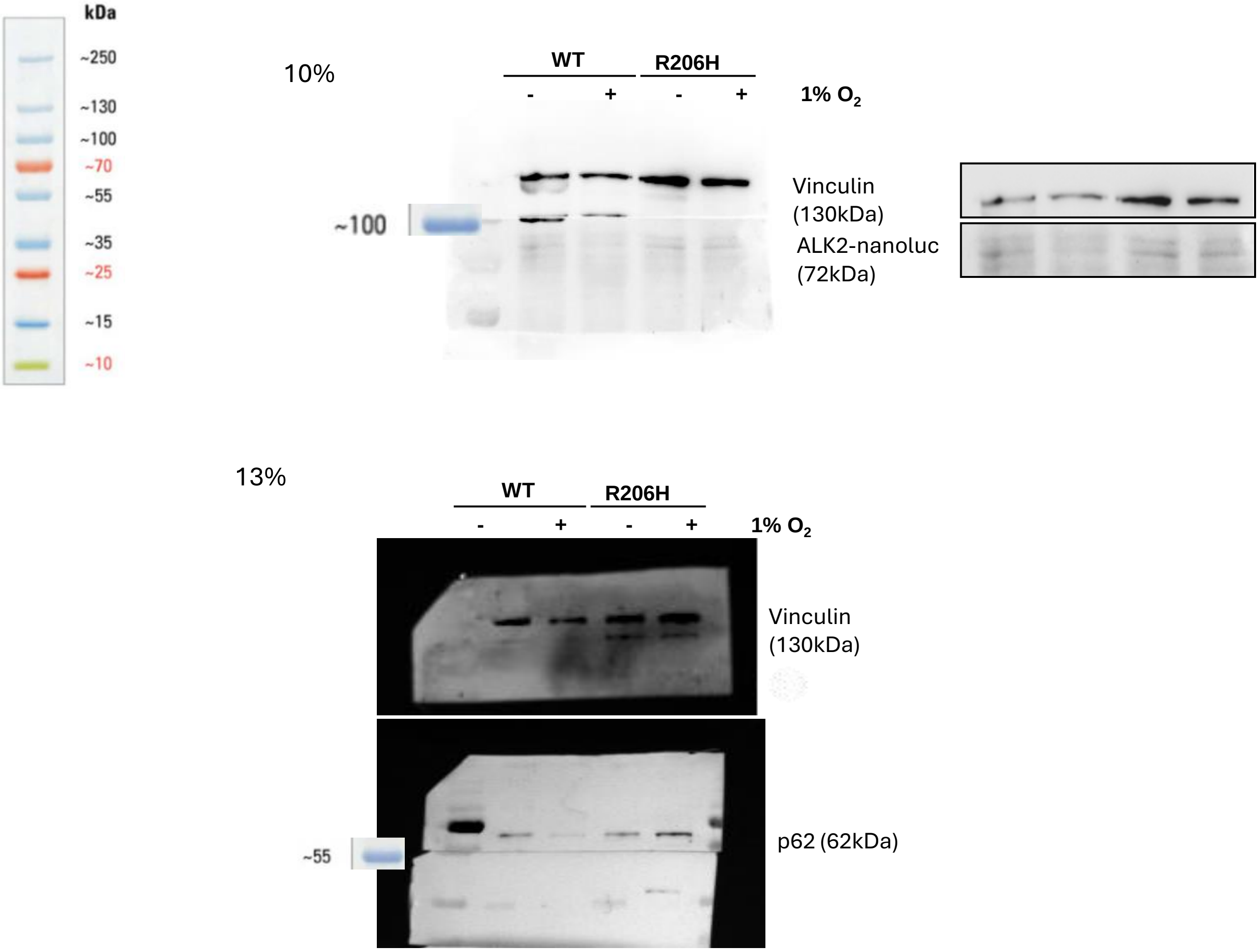

Figure 2D

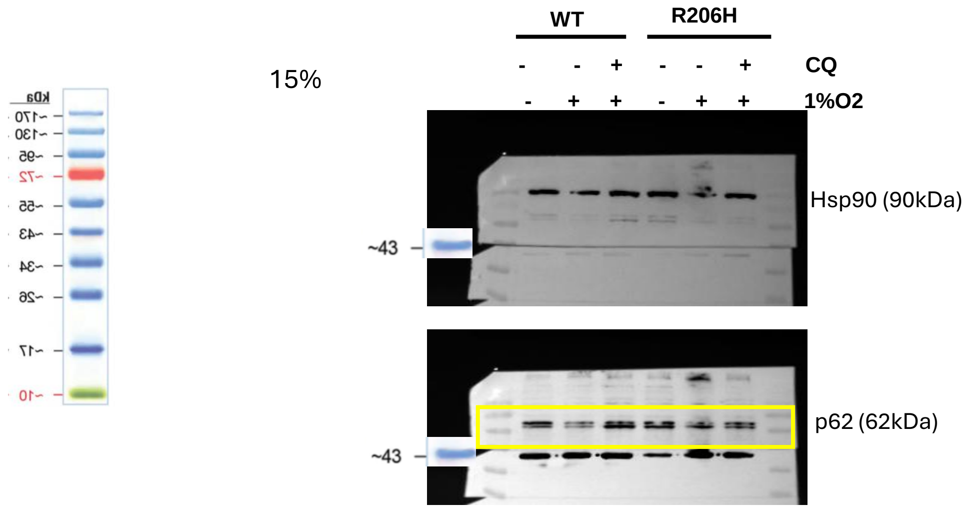

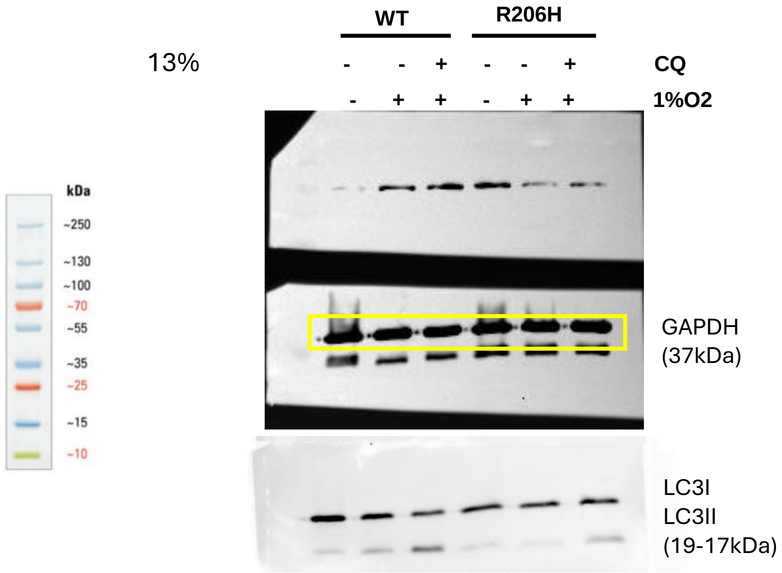

Figure 2E

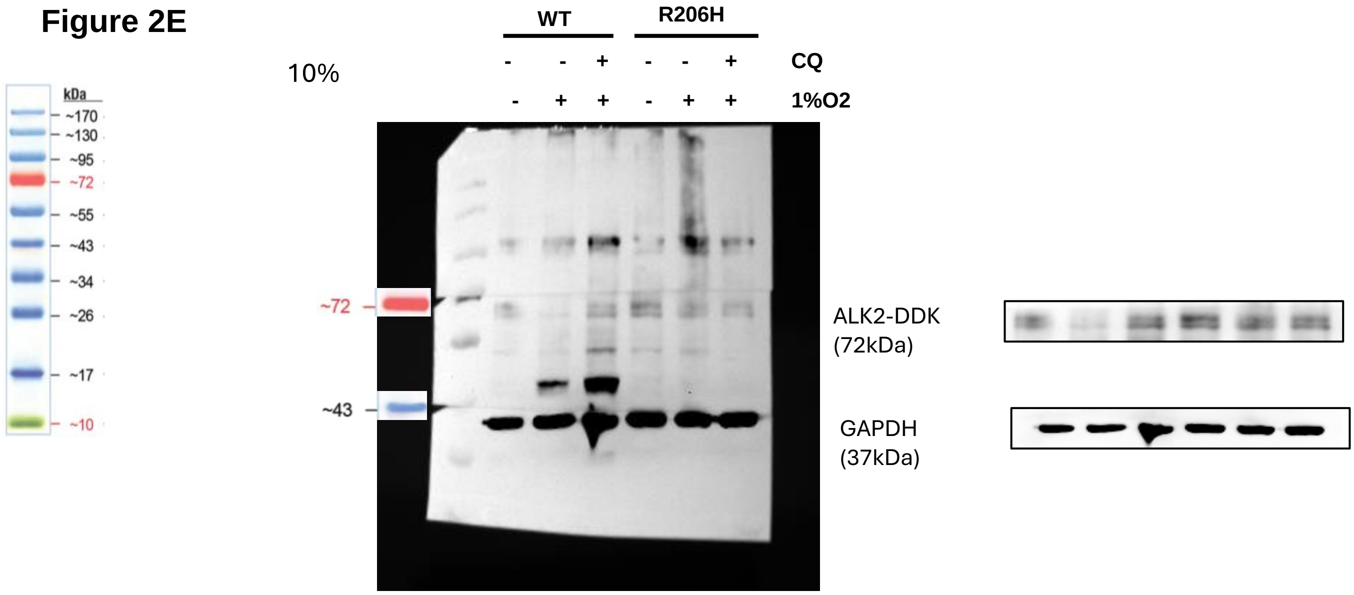

Figure 3B

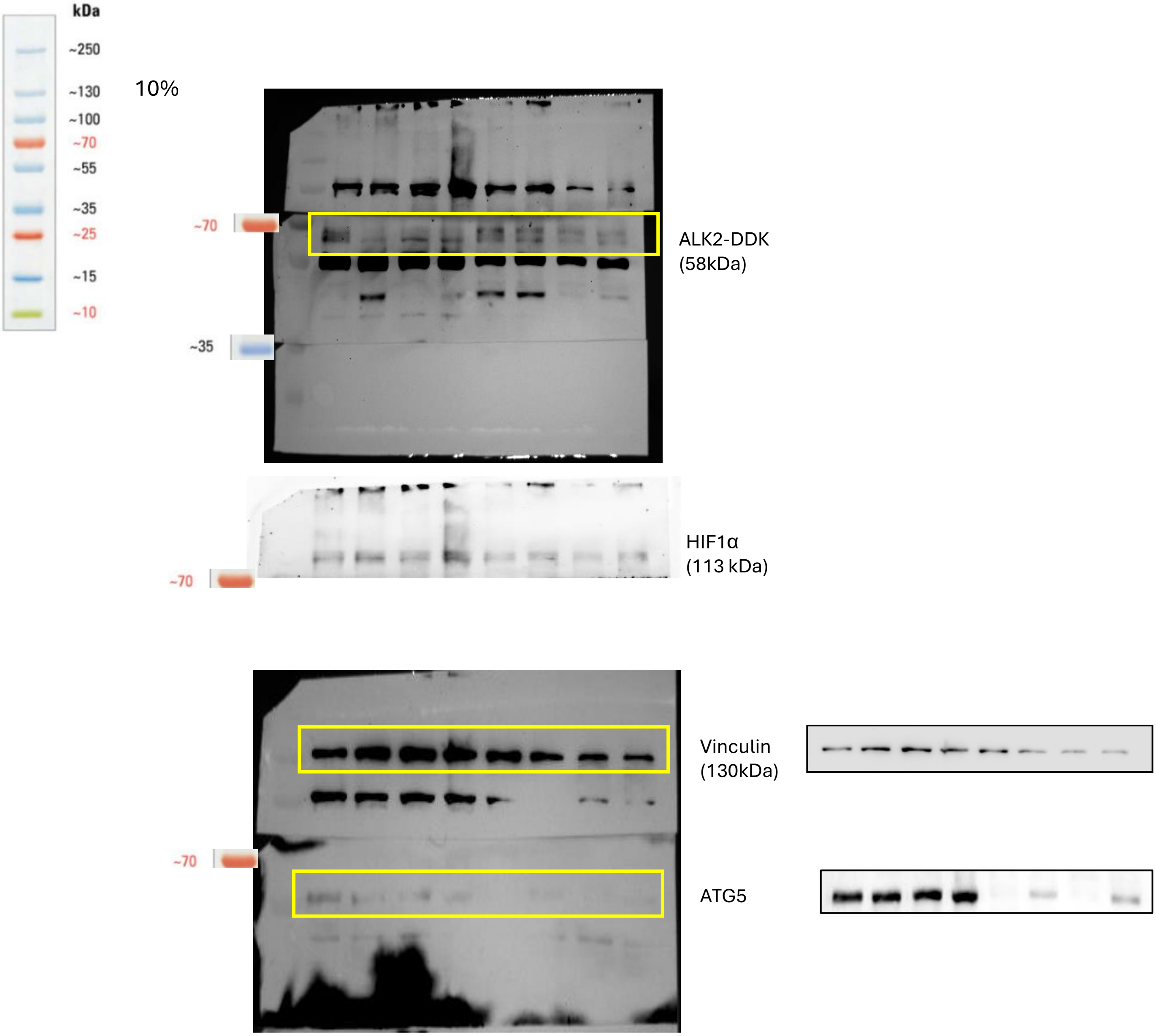
kDa
~250
~130
~100
~70
~55
~35
~25
~15
~10
10%
~70
~35
ALK2-DDK
(58kDa)
~70
HIF1α
(113 kDa)
Vinculin
(130kDa)
~70
ATG5

Figure 4A

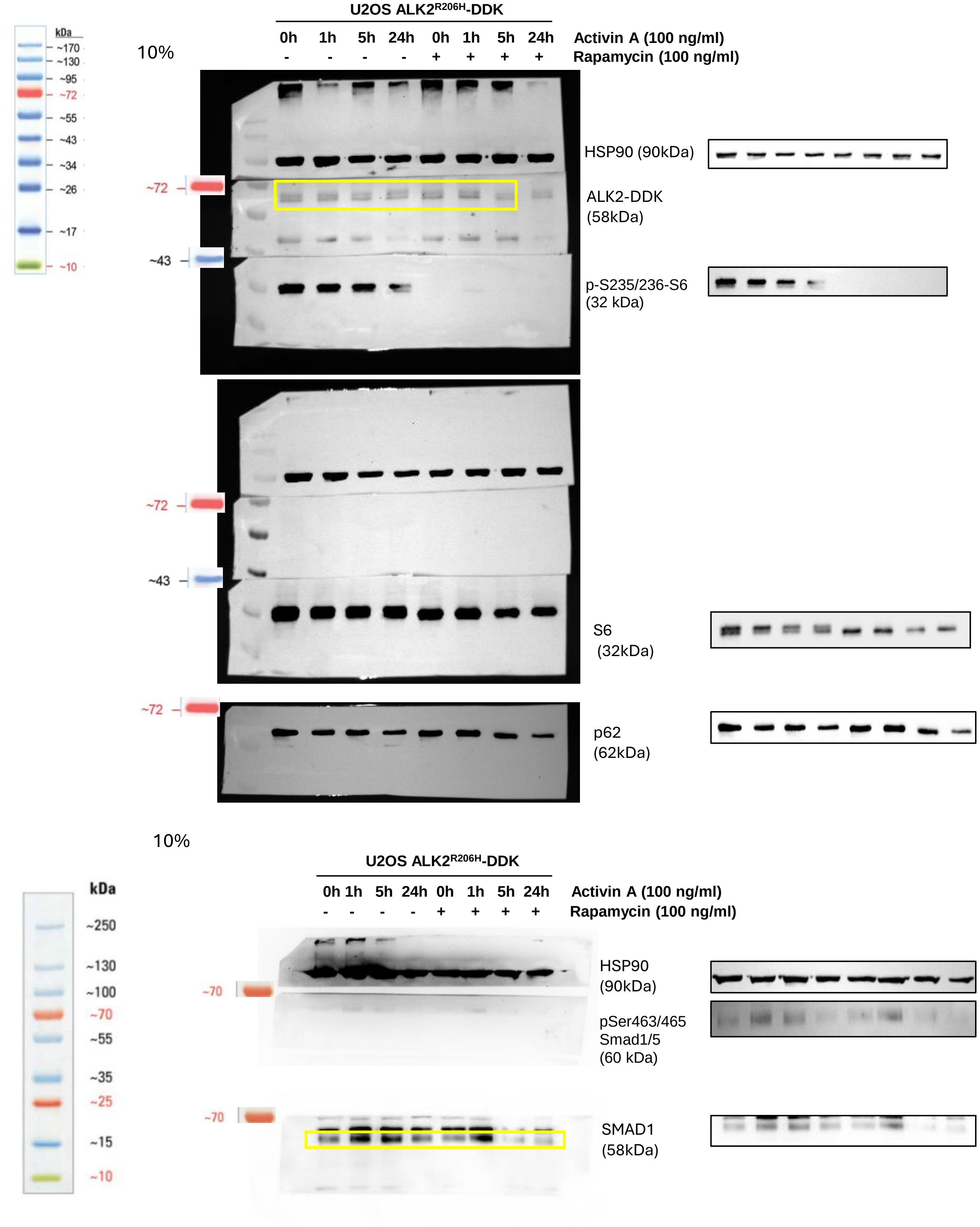

U2OS ALK2R206H-DDK
10%
0h 1h 5h 24h 0h 1h 5h 24h
Activin A (100 ng/ml)
Rapamycin (100 ng/ml)
kDa
~170
~130
~95
~72
~55
~43
~34
~26
~17
~10
HSP90 (90kDa)
ALK2-DDK
(58kDa)
p-S235/236-S6
(32 kDa)
S6
(32kDa)
p62
(62kDa)
10%
U2OS ALK2R206H-DDK
0h 1h 5h 24h 0h 1h 5h 24h
Activin A (100 ng/ml)
Rapamycin (100 ng/ml)
kDa
~250
~130
~100
~70
~55
~35
~25
~15
~10
HSP90
(90kDa)
pSer463/465
Smad1/5
(60 kDa)
SMAD1
(58kDa)

Figure 5A Figure 5B

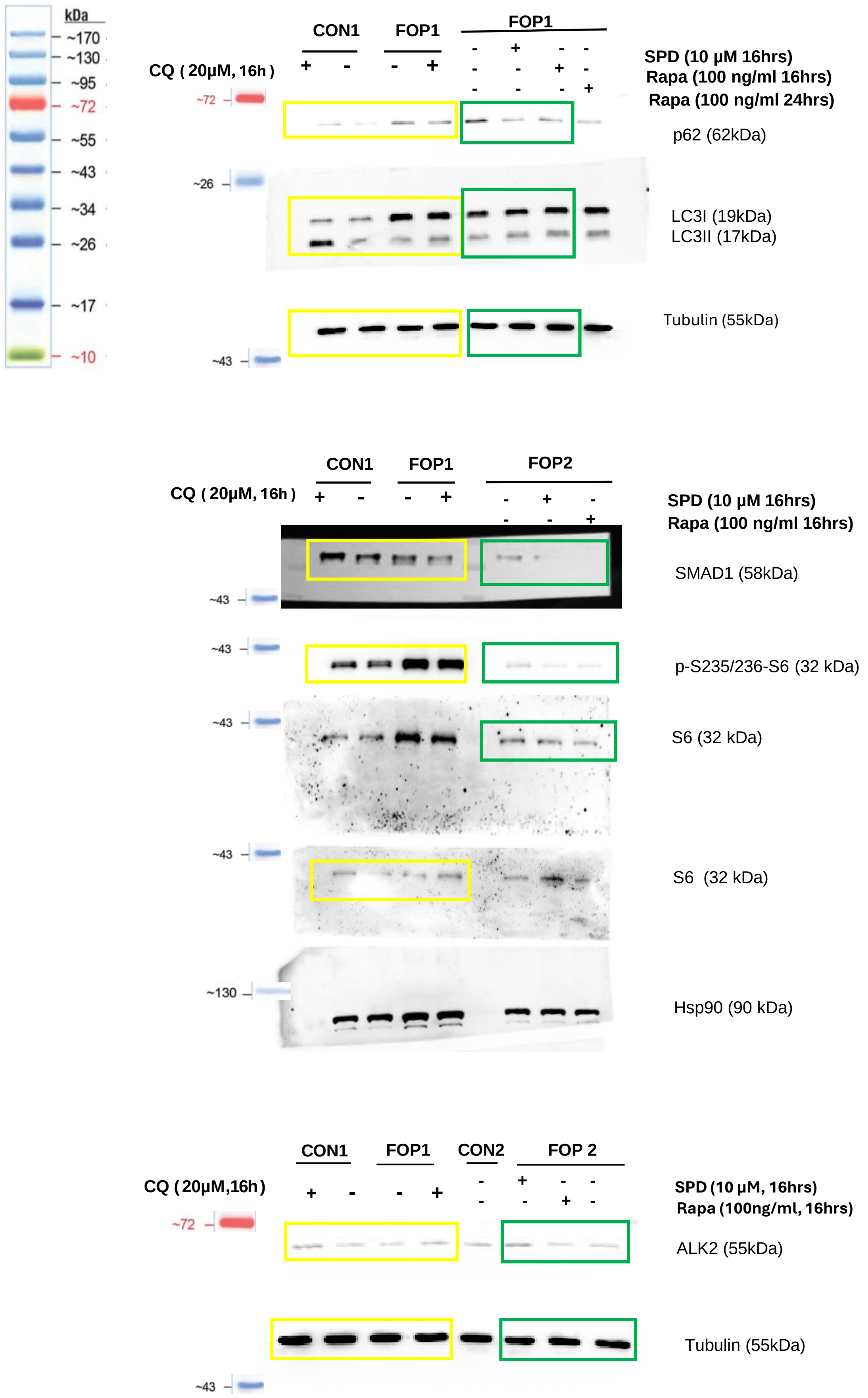

Figure S1 A

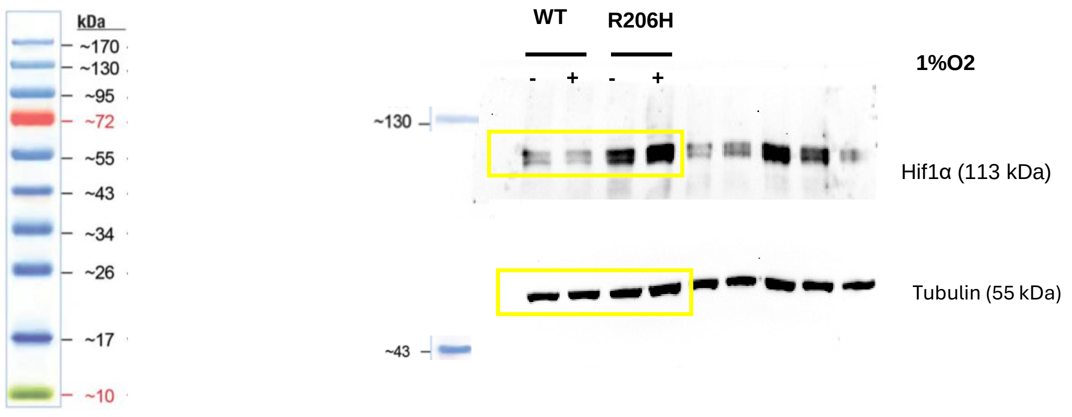

Figure S1 C

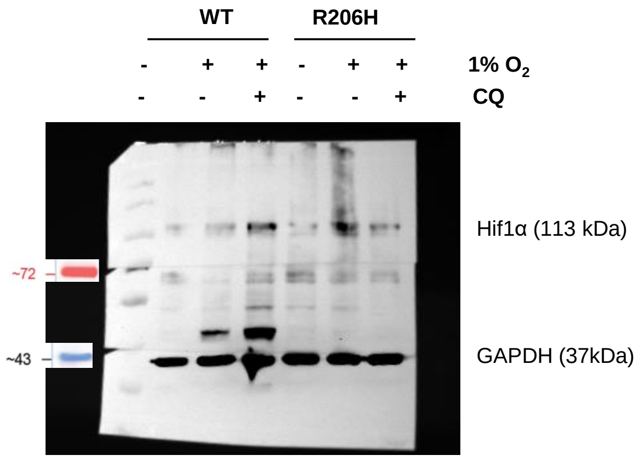

Figure S1 D

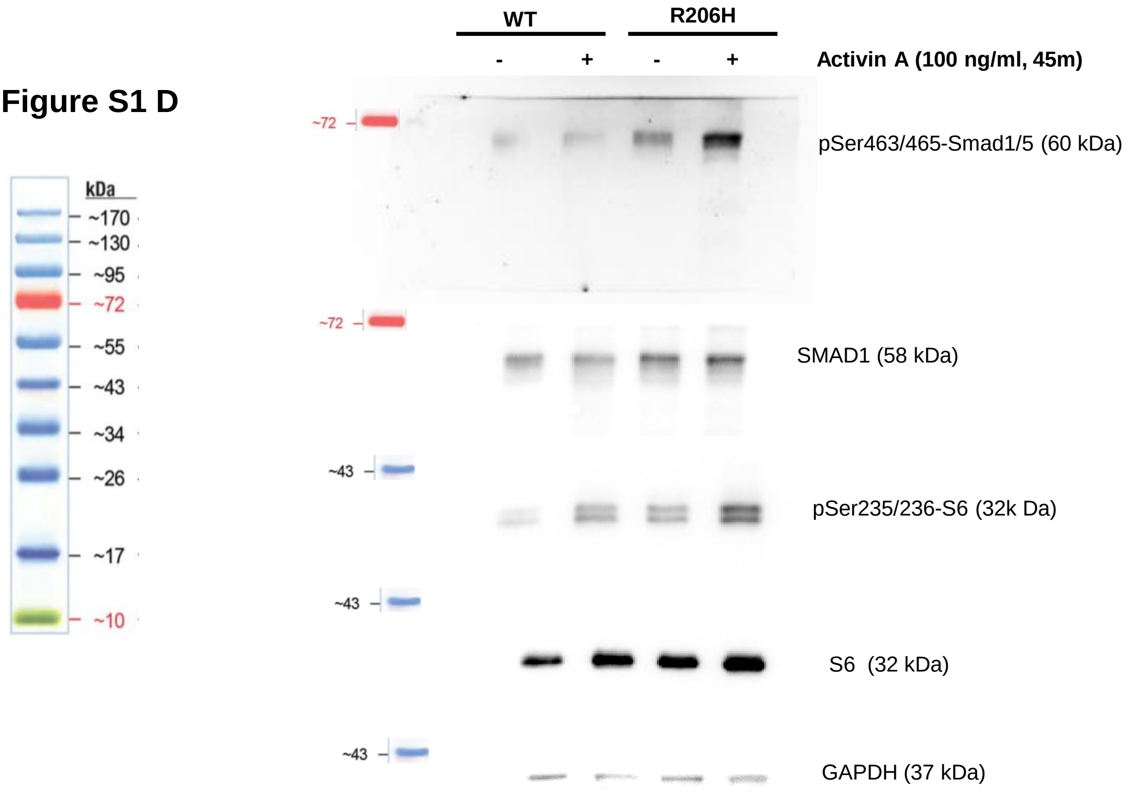

Figure S2 A

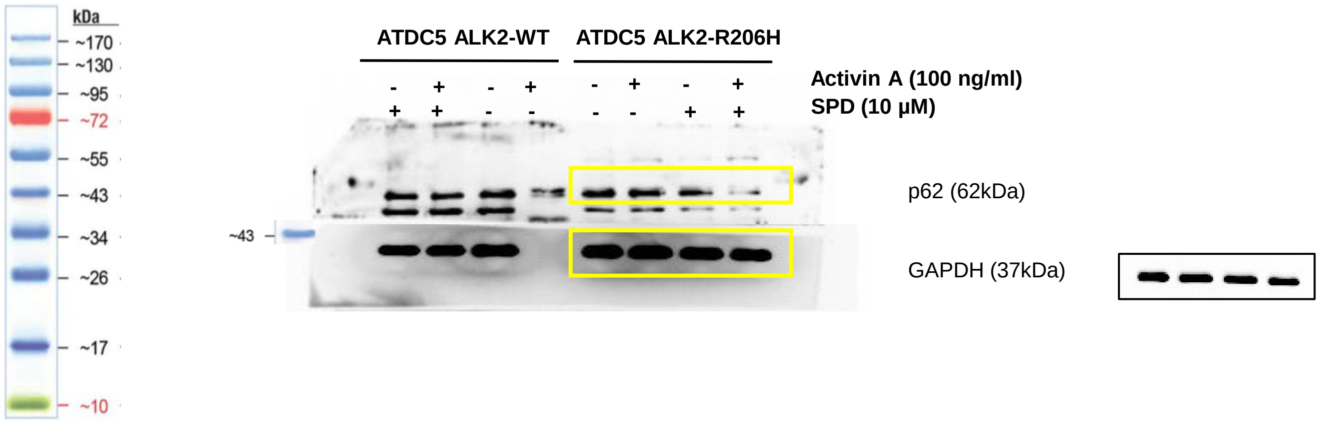

Figure S2 D

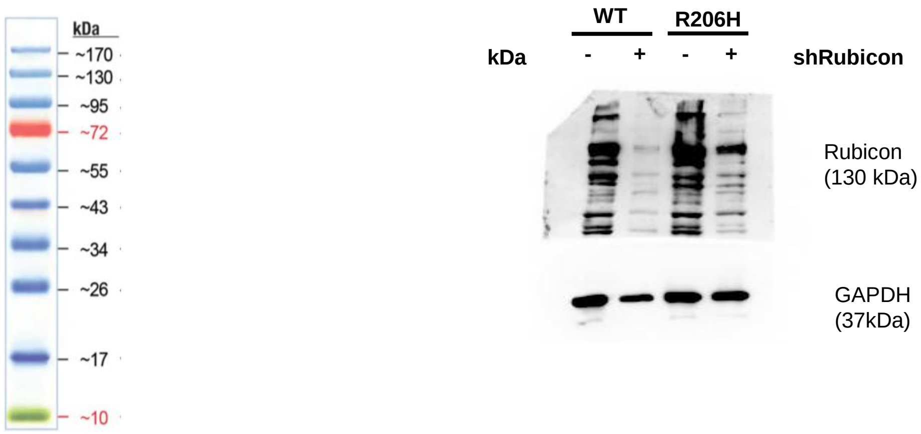

Supplement: Supplementary file 4 — Original data [file 41420_2025_2393_MOESM4_ESM.pdf]
